# A computational method to quantitatively measure pediatric drug safety using electronic medical records

**DOI:** 10.1186/s12874-020-0902-x

**Published:** 2020-01-14

**Authors:** Gang Yu, Xian Zeng, Shaoqing Ni, Zheng Jia, Weihong Chen, Xudong Lu, Jiye An, Huilong Duan, Qiang Shu, Haomin Li

**Affiliations:** 1 The Children’s Hospital of Zhejiang University School of Medicine and National Clinical Research Center for Child Health, Hangzhou, China; 2 College of Biomedical Engineering and Instrument Science, Zhejiang University, Hangzhou, China; 3 Department of Pharmacy, Shanxi Dayi Hospital, Taiyuan, China

**Keywords:** Pediatric drug safety, Electronic medical record, Drug clustering, Quantitative measurement

## Abstract

**Background:**

Drug safety in children is a major concern; however, there is still a lack of methods for quantitatively measuring, let alone to improving, drug safety in children under different clinical conditions. To assess pediatric drug safety under different clinical conditions, a computational method based on Electronic Medical Record (EMR) datasets was proposed.

**Methods:**

In this study, a computational method was designed to extract the significant drug-diagnosis associations (based on a Bonferroni-adjusted hypergeometric *P*-value < 0.05) among drug and diagnosis co-occurrence in EMR datasets. This allows for differences between pediatric and adult drug use to be compared based on different EMR datasets. The drug-diagnosis associations were further used to generate drug clusters under specific clinical conditions using unsupervised clustering. A 5-layer quantitative pediatric drug safety level was proposed based on the drug safety statement of the pediatric labeling of each drug. Therefore, the drug safety levels under different pediatric clinical conditions were calculated. Two EMR datasets from a 1900-bed children’s hospital and a 2000-bed general hospital were used to test this method.

**Results:**

The comparison between the children’s hospital and the general hospital showed unique features of pediatric drug use and identified the drug treatment gap between children and adults. In total, 591 drugs were used in the children’s hospital; 18 drug clusters that were associated with certain clinical conditions were generated based on our method; and the quantitative drug safety levels of each drug cluster (under different clinical conditions) were calculated, analyzed, and visualized.

**Conclusion:**

With this method, quantitative drug safety levels under certain clinical conditions in pediatric patients can be evaluated and compared. If there are longitudinal data, improvements can also be measured. This method has the potential to be used in many population-level, health data-based drug safety studies.

## Background

Although children differ from adults in many aspects of pharmacotherapy, children are often excluded from clinical trials, and knowledge about the efficacy and safety of drugs used in this population is scarce. This lack of knowledge often results in frequent unlicensed/off-label drug use in children. In 2004, the EMA (European Medicines Agency) published a report on the evidence of harm associated with the use of unlicensed and off-label drug use in children [1]. However, few studies have evaluated general drug use in children based on two literature reviews [2, 3]. Some of the population-based studies provide trends of outpatient drug use [4, 5]. A few studies focus on hospitalized children [6]. Several other studies have concentrated on a limited number of drugs, such as antibiotics and psychotropic drugs [7, 8]. However, there are no clinical tools or methods that could quantitatively measure drug safety under different pediatric conditions that can be used to balance priorities between common drugs with wide-reaching effects (such as asthma medications) and rarer drugs with smaller target populations or more serious consequences (such as cardiovascular drugs).

Under the Best Pharmaceuticals for Children Act in the US, the National Institute of Child Health and Human Development (NICHD) is responsible for annually updating a priority list of drugs that require further study in children. Currently, such a task requires intensive expertise and data analysis to identify clinical scenarios with high priority for drug safety. Meanwhile, the results may not be widely accepted due to a lack of objectivity. Some opinion leaders have advocated the prioritization of pediatric drug research using population-level health data and have proposed to address 5 key considerations: the prevalence of disease, patterns of drug use, adverse drug events, drug-drug interactions, and drugs with the potential for misuse [9]. To address the above five concerns, Electronic Medical Records (EMRs) are one of the best resources. Due to the promotion of EMR systems worldwide, increasing amounts of clinical data have been accumulated electronically and are ready for use [10]. Real-world clinical data, which imply knowledge of both diseases and clinical drug utilization, concomitantly provide drug and diagnostic relationships under certain clinical scenarios [11]. Furthermore, comparing real-world clinical data could provide information about drugs that should be used but not included in pediatrics due to safety concerns. Here, we propose a computational method that compares pediatric drug use patterns and measures their safety under specific clinical conditions. Based on this approach, the pediatric drug safety under different clinical conditions can be measured based on objective data and addressed the major concerns in this field.

## Methods

**Fig. 1:**
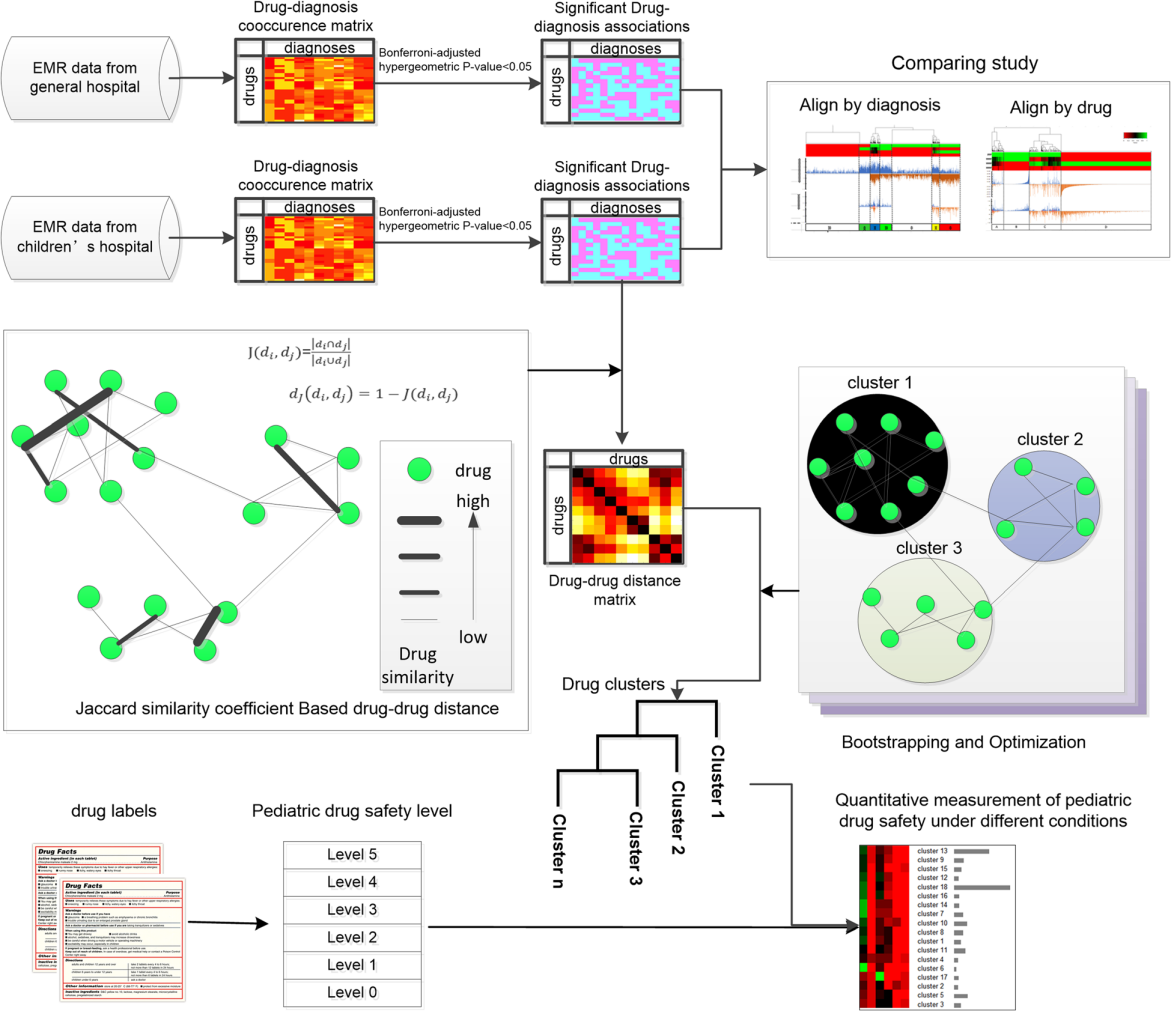
Workflow of the computational method

In this study, a computational method was designed to extract significant drug-diagnosis associations from two EMR datasets, one generated in a children’s hospital and the other in a general hospital. By comparing the drug-diagnosis associations by aligning them with the drugs used and the diagnoses made in each hospital, drug treatment gaps between the children’s hospital and the general hospital can be identified and visualized. With the exception of comparing the differences between the two datasets, the drug-diagnosis associations were used to create drug clusters under specific clinical conditions using unsupervised clustering based on drug-drug distances. A definition of pediatric drug safety level was proposed based on the drug safety statement on its pediatric labeling. Therefore, quantitative measurements of pediatric drug safety levels under different clinical conditions can be compared. This quantitative information can be used to improve safety and healthcare decision making. It has the potential to be used in many population-level, health data-based drug use studies. The workflow of this computational method is shown in Fig. 1.

### Constructing significant drug-diagnosis associations from EMRs

The drug and diagnosis codes under the same inpatient encounter can be used to generate a drug-diagnosis association pair. A drug-diagnosis co-occurrence matrix was created based on the number of drug-diagnosis association pairs in the whole dataset. Using this drug-diagnosis co-occurrence matrix, we calculated a hypergeometric *P*-value using the *phyper* function in the *stats* package under R (version 3.4.0). After this enrichment, Bonferroni correction was conducted to control the family-wise error rate for multiple testing [12]. All drug-diagnosis associations with Bonferroni-adjusted *P* < 0.05 were considered statistically significant.

Significant drug-diagnosis associations can be used to compare the differences between pediatric drug use and general adult drug use. Hierarchical cluster analysis (*hclust* function in the *stats* package of R version 3.4.0) was used to identify different diagnosis groups and drug groups. A heatmap was used to visualize the features of different groups. The ICD-10 codes (International Classification of Disease, 10th edition) for diagnosis and ATC (Anatomical Therapeutic Chemical) codes for drugs were used to group and enrich these features.

### Measuring drug-drug distances and constructing drug clusters

The drug-drug distance was measured based on the Jaccard similarity coefficient, which is performed on the significant drug-diagnosis association matrix, as shown in formula 1. The *P*-values in the enriched matrix were converted to binary bits, where *P* < 0.05 was set to 1 and other items were set to 0.

1 $$ {d}_J\left({d}_i,{d}_j\right)=1-\frac{\left|{d}_i\cap {d}_j\right|}{\left|{d}_i\cup {d}_j\right|} $$

To cluster the drugs used in the children’s hospital based on the drug-drug distance, the k-means clustering algorithm was chosen because of its unsupervised feature and ability to handle large datasets [13]. A bootstrap strategy was used to select the best k from 10 to 50. The *clusterboot* function from the *fpc* package in R was used for the above method. Source code in R for all above methods was available in the supplemental materials.

### Defining the drug safety level in children

Currently, there are no quantitative drug safety levels defined. In this study, a quantitative drug safety level in children was defined based on the classification of the pediatric population, as shown in Table 1. If the drug labels contained statements such as “the safety and efficacy in pediatric patients have not been established”, the drug safety level in children was set at level 0. The minimum age range that was indicated in pediatric labeling was used to set the safety level. For example, the safety level of *amlodipine besylate tablets* was set to level 2, as its pediatric labeling shows that its recommended dosages are for children from 6 to 17 years old. If there was only a general indication of its use in children without a clear age range, the drug safety level was set to level 2 by default. All 591 drugs used in the children’s hospital were manually assigned a drug safety level based on their pediatric labels. The drug safety levels of drug clusters were calculated by averaging the drug safety levels of each drug in each cluster. Therefore, pediatric drug safety under different clinical conditions can be measured and compared.

**Table 1:** Defining pediatric drug safety level

Safety Level	Drug safely used in pediatric population
5	Preterm newborn infants
4	Full-term newborn infants (0 to 28 days)
3	Infants and toddlers (> 28 days to 23 months)
2	Children (2 to 11 years) or applicable in children without specific age statement
1	Adolescents (12 to 18 years)
0	Without pediatric labeling/contraindicated or not applicable in children

### EMR datasets from two hospitals

An EMR system that was implemented in a 1900-bed children’s hospital (Children’s Hospital, Zhejiang University School of Medicine) was used to construct this drug-diagnosis associations dataset. Total of 869,833 medication records and 129,829 diagnosis codes were extracted from 64,186 encounters of 56,235 patients who were admitted to the hospital between January 1, 2016 and December 31, 2016. The system generated 1,667,573 drug-diagnosis association pairs that contained 591 distinct drugs and 3207 distinct diagnosis codes. After enrichment (see details in the Methods section), only 7269 statistically significant drug-diagnosis associations were retained.

The other EMR system, implemented in a 2000-bed general hospital (Shanxi Dayi Hospital), was used to construct the other drug-diagnosis association dataset. A total of 812,554 medications and 339,269 discharge diagnosis codes were extracted from 53,922 encounters of 46,255 patients who were admitted to the hospital between January 1, 2016 and December 31, 2016. The system generated 6,039,728 drug-diagnosis association pairs that contained 1210 distinct drugs and 6901 distinct diagnosis codes. After enrichment, a total of 20,297 statistically significant drug-diagnosis pairs were retained.

This study was approved by the Institutional Review Board/Ethics Committee of Children’s Hospital of Zhejiang University School of Medicine (Hangzhou, China) and signed data use agreement was obtained for the dataset from Shanxi Dayi Hospital.

## Results

### The drug treatment gap between the children’s hospital and the general hospital

The two significant drug-diagnosis association datasets, generated from the children’s hospital and the general hospital, can be compared by performing alignments by diagnoses and by drugs.

### Alignment by diagnoses

The disease spectrum of inpatient children was different from that of inpatient adults, as shown in Additional file 1: Figure S1. The flourishing of congenital malformations and conditions originating in the perinatal period in the children’s hospital is undoubtedly logical. Diseases of the respiratory system are the most common conditions in the children’s hospital. The counterpart in the general hospital is diseases of the circulatory system. Another prevailing disease class in the children’s hospital is infectious and parasitic diseases. Injuries, poisoning and other consequences of external causes are also relatively higher in children than in adults. At the same time, two hospitals also share many diseases.

**Fig. 2:**
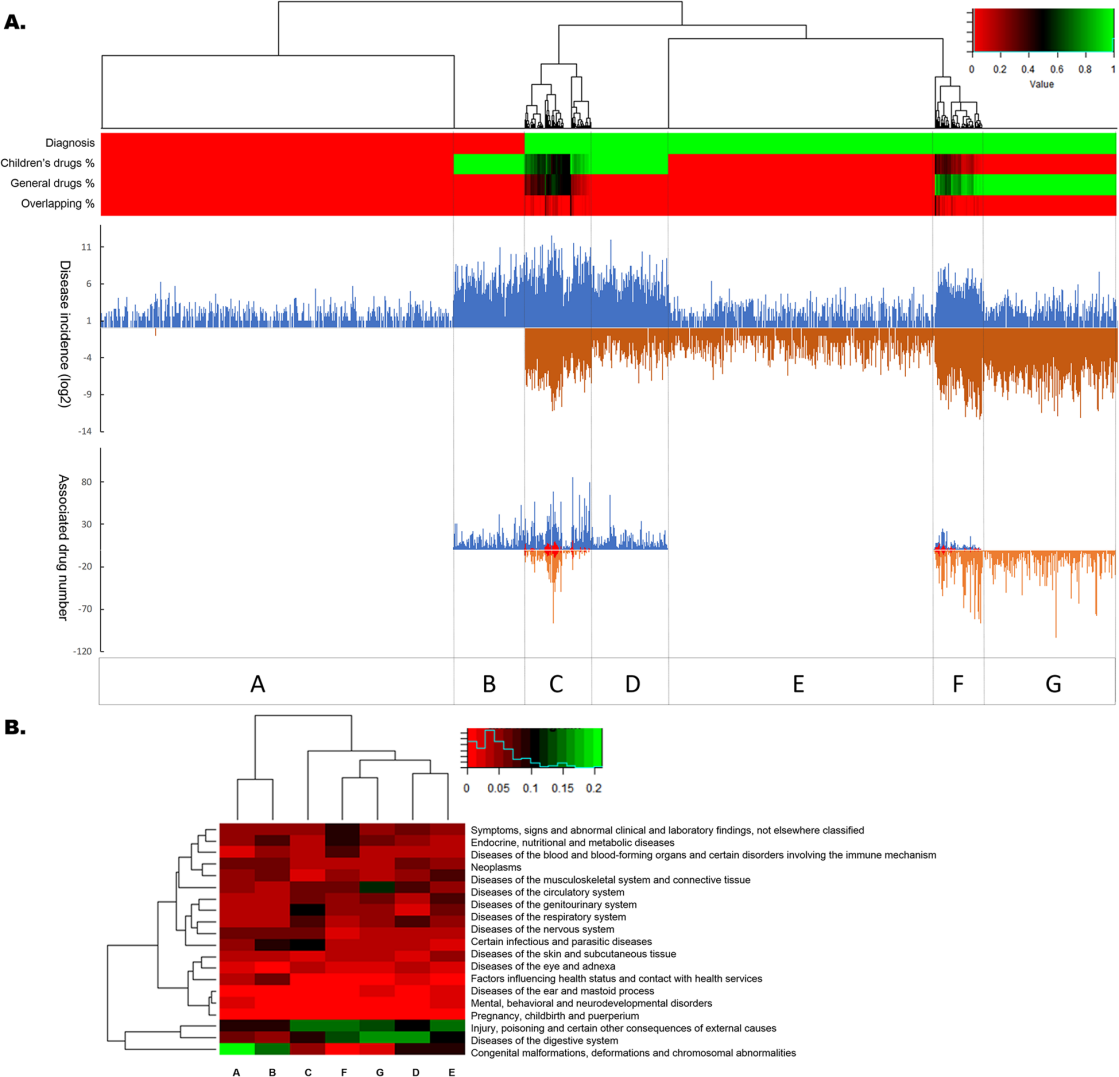
Comparing drug use in children and adults aligned by diagnosis. **a**. The heatmap on the top section shows the clustering of the diagnoses. The first row of the heatmap shows the 3205 diagnoses used in the children’s hospital. The red section contains 1137 unique diagnoses only used in the children’s hospital. The other 2068 diagnoses shown in green are also used in the general hospital. The other three rows of the heatmap show, from top to bottom, the percentage of associated drugs in the children’s hospital, the general hospital, and their overlap. The mirror histograms in the middle show the incidences of these diagnoses in the children’s hospital (blue) and the general hospital (brown). The bottom mirror histograms show the number of associated drugs in the children’s hospital (blue), the general hospital (brown) and their overlap (red). Based on the clustering, the disease spectrum in the children’s hospital can be classified into groups A-G (7 groups). **b**. The heatmap shows the percentages of ICD-10 categories belonging to the 7 (A-G) groups generated in Fig. 2a

All 3205 diagnoses that were used in the children’s hospital, including 1137 unique diagnoses only used in the children’s hospital, were used to align the drug-diagnosis associations (shown in Fig. 2). The disease spectrum was divided into groups A through G (7 groups). Group A contains diagnoses only used in the children’s hospital and with low incidence. Group B also contains diseases unique to the children’s hospital but with relatively high incidence, as well as associated drugs. Group C contains diseases with high incidence and associated drugs in both the children’s hospital and the general hospital. However, the associated drugs for this group are more frequently used in children’s hospitals. Group D contains diseases with a high incidence in the children’s hospital and a relatively low incidence in the general hospital. Diseases in group D have associated drugs in the children’s hospital but no associated drugs in the general hospital. Group E contains low-incidence diseases in both hospitals and no associated drugs in both hospitals. Group F is similar to group C. However, the associated drugs are more frequently used in the general hospital. Group G contains diseases with a high incidence and associated drugs in the general hospital but with low incidence and no associated drugs in the children’s hospital.

For different disease groups, there are distinctive concerns regarding drug use in children. As shown in Fig. 2b, congenital malformations are relatively major components of groups A and B. Groups C and F, which had the best overlap in drug use between the children’s hospital and the general hospital, focused on injuries, poisoning and other consequences of external causes. The difference between groups D and G, which showed distinct drug features in the two hospitals, is reflected by the different incidences of diseases of the circulatory system and respiratory system and of congenital malformations in the two hospitals. For groups A, E and G, the concern was why there were no associated drugs in the children’s hospital. One reason is the low incidence of these diseases (average incidence in inpatients was 0.059‰ in group A, 0.062‰ in group E, and 0.078‰ in group G) and another reason may due to the concerns about drug safety and do not allow these drugs used in pediatrics. For groups B, C, D and F, which have associated drugs in the children’s hospital, the concern was whether those drugs were safe to use in children.

### Alignment by drugs

**Fig. 3:**
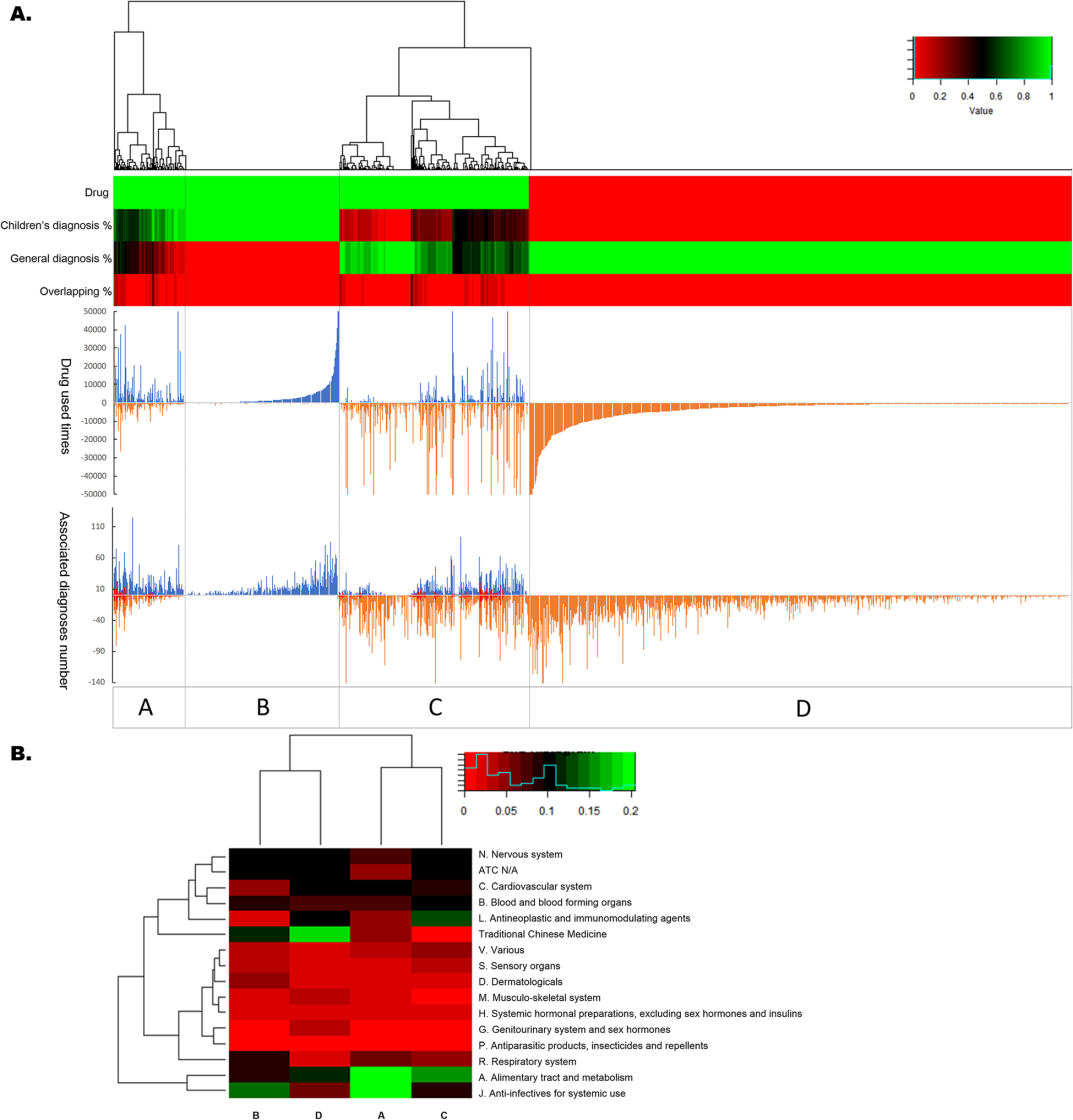
Comparing drug use in children and adults aligned by drug. **a**. The heatmap on the top section shows the clustering of the drugs. The first row of the heatmap shows the 1185 drugs that are associated with at least one diagnosis in either the children’s hospital or the general hospital. The green section contains 513 drugs used in the children’s hospital. The other 672 drugs shown in red were only used in the general hospital. The other three rows of the heatmap show, from top to bottom, the percentage of associated diagnoses in the children’s hospital, the general hospital, and their overlap. The mirror histograms in the middle show the volume of these drugs used in the children’s hospital (blue) and the general hospital (brown). The bottom mirror histograms show the number of associated diagnoses in the children’s hospital (blue), the general hospital (brown) and their overlap (red). Based on the clustering, the drugs can be classified into groups A-D (4 groups). **b**. The components of the A-D drug groups generated in Fig. 3a. The percentages of ATC first-level codes (including two special drug categories: drugs without an assigned ATC code and drugs classified as TCM) in each group are plotted in the heatmap

There are 591 distinct drugs used in the children’s hospital and 1210 in the general hospital. A total of 1185 drugs that were associated with at least one diagnosis in one of the two hospitals were used to align the drug-diagnosis associations in Fig. 3a. All drugs were divided into groups A to D (4 groups). Group A drugs were associated with diagnoses in the two hospitals, with associated diagnoses in the children’s hospital prevailing over those in the general hospital. Drugs in group B were mainly used in the children’s hospital and without associated diagnoses in the general hospital. Group C was similar to group A, but the associated diagnoses in the general hospital were dominant. Drugs in group D were only used in the general hospital.

The components of the different types of drugs based on the ATC category in drug groups A through D are presented in Fig. 3b. For group A, the anti-infectives and drugs related to the alimentary tract and metabolism were highlighted. The predominance of traditional Chinese medicine (TCM) in two distinct groups, group B and group D, shows that TCM still plays a very important role in all populations in China. Although most TCM does not have pediatric labeling, clinicians and parents like to seek TCM treatment if there are no drugs or if there are drugs with side effects that are not tolerated by children. A total of 50 (8.46%) types of TCM were used in the children’s hospital. Most of these types of TCM are in child-friendly forms, such as oral liquids or granules. In group C, the alimentary tract/metabolism-related drugs and antineoplastic/immunomodulating agents are highlighted. The relatively low rate of anti-infectives in group D also shows the differences in the disease spectrum between children and adults. Except for the relatively high incidence of infectious and parasitic diseases in the children’s hospital, the number one disease category in children is diseases of the respiratory system, which are usually also caused by infectious diseases.

### Drug clusters under pediatric clinical conditions

**Fig. 4:**
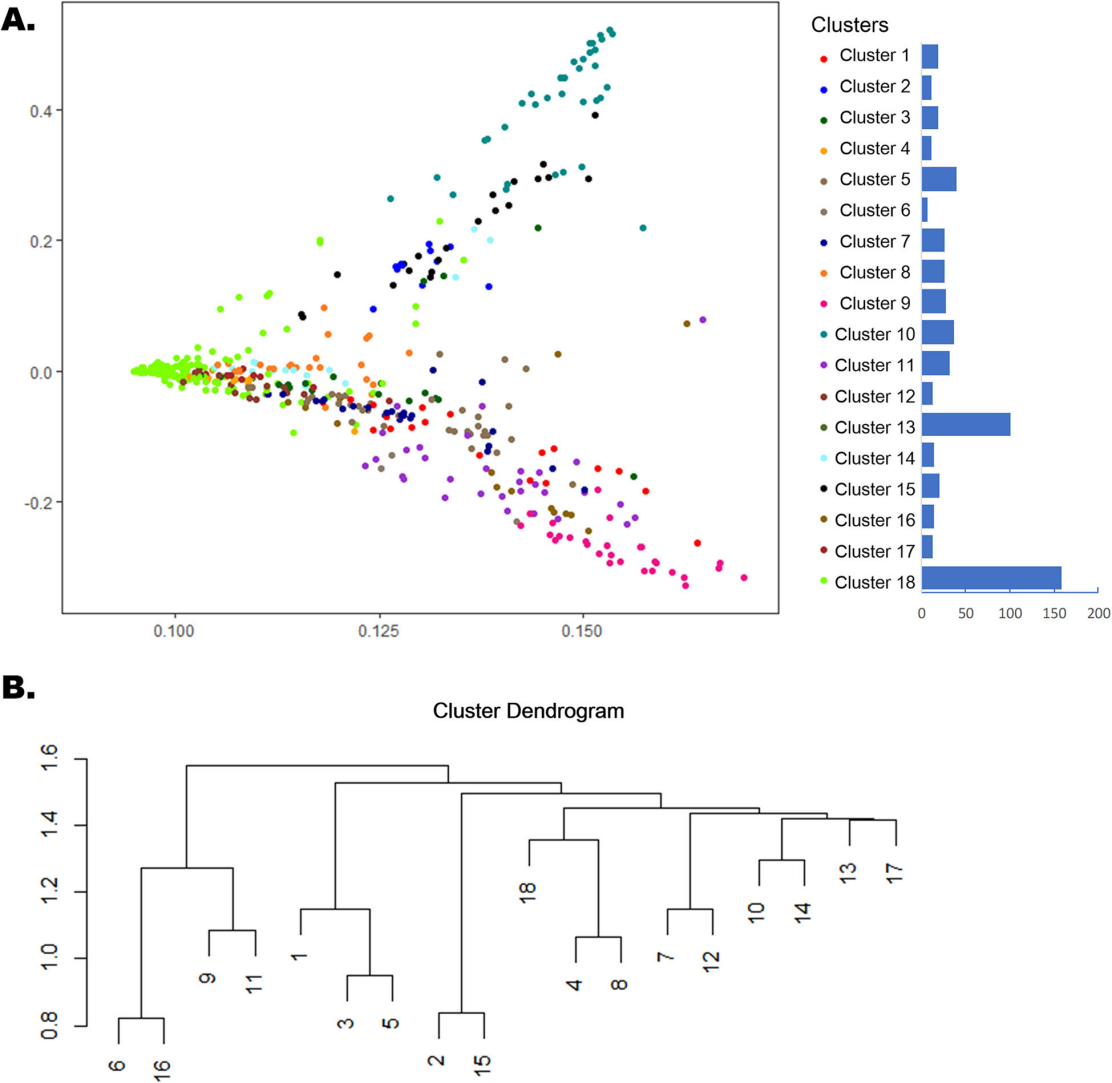
Drug clusters under different clinical conditions in the children’s hospital. **a**. Plot of multidimensional scaling (MDS) of the drug-drug distance matrix in 2D colored by cluster (cluster 13, an outlier, is not shown in this view). The size of each drug cluster is plotted as bar chart on the right side. **b**. The hierarchy of the 18 drug clusters is measured based on the distance of the associated diagnosis sets for each drug cluster

During clustering of the drugs in the children’s hospital, the mean Jaccard index of each k-means clustering showed the highest value when k was set to 18. Then, we obtained 18 drug clusters from the 519 drugs used in the children’s hospital, as shown in Fig. 4. Cluster 13 is a special cluster that contains drugs that are not associated with any diagnosis and are not frequently used in clinical practice (the drug use volumes of each drug cluster are shown in Additional file 1: Figure S2). Cluster 18 contains drugs located in the densest part, in which drugs are difficult to discriminate due to their infrequent use and lack of association with many problems. The other 16 drug clusters were separated and used under particular clinical scenarios. Details about the kernel diagnoses of each drug cluster are shown in Additional file 1: Table S1. A website was developed to help in the exploration of these clusters. Please access http://kb4md.org:4000/peddrugcluster and select the cluster number on the webpage, which will show the distribution of the clustered drugs for all drugs, the hierarchical structure of the drugs in the cluster, the similarity of each drug, and the associated diagnosis codes and the ATC classes of this cluster.

These 18 drug clusters were hierarchically clustered based on the distances between their kernel diagnosis codes (as shown in Fig. 4b). Clusters 6 and 16 are associated with acute lymphoblastic leukemia (ALL) and other cancers respectively. Clusters 9 and 11 are involved in the treatment of ALL-related infectious conditions and convalescence following problems. Clusters 1, 3 and 5 are all associated with diseases of the respiratory system. Cluster 5 focuses on pneumonia, cluster 1 focuses on acute upper respiratory infection, and cluster 3 focuses on other diseases of the upper respiratory tract. Clusters 2 and 15 are related to preterm and full-term newborn problems, respectively. Cluster 4 is related to diseases of the digestive system, such as chronic superficial gastritis. Cluster 8 is related to common pediatric surgery problems, such as adherent prepuce and acute appendicitis, which also belong to diseases of the digestive system. Clusters 7 and 12 focus on nephrotic syndrome and chronic kidney disease, respectively. Other clusters are relatively independent of each other. Cluster 10 is related to congenital heart diseases, and cluster 14 is related to cerebral contusions. Cluster 17 is specifically associated with epilepsy.

All these clinical scenarios of drug clusters ware objective and do not depend on experts. As you can see from the Additional file 1: Table S1, the myocardial injury was show up in the pneumonia cluster 5 due to it is the major and severe complications of pneumonia [14, 15]. The acute upper respiratory infection was show up in the kidney disease cluster because it is proved that at least 50% of activities in pediatric onset Nephrotic Syndrome are stimulated by a viral upper respiratory tract infection [16, 17]. These superficial inconsistencies reflect the real-world clinical scenarios, and it also show exactly the advantage of this computational methods.

### Drug safety level in inpatient children

**Fig. 5:**
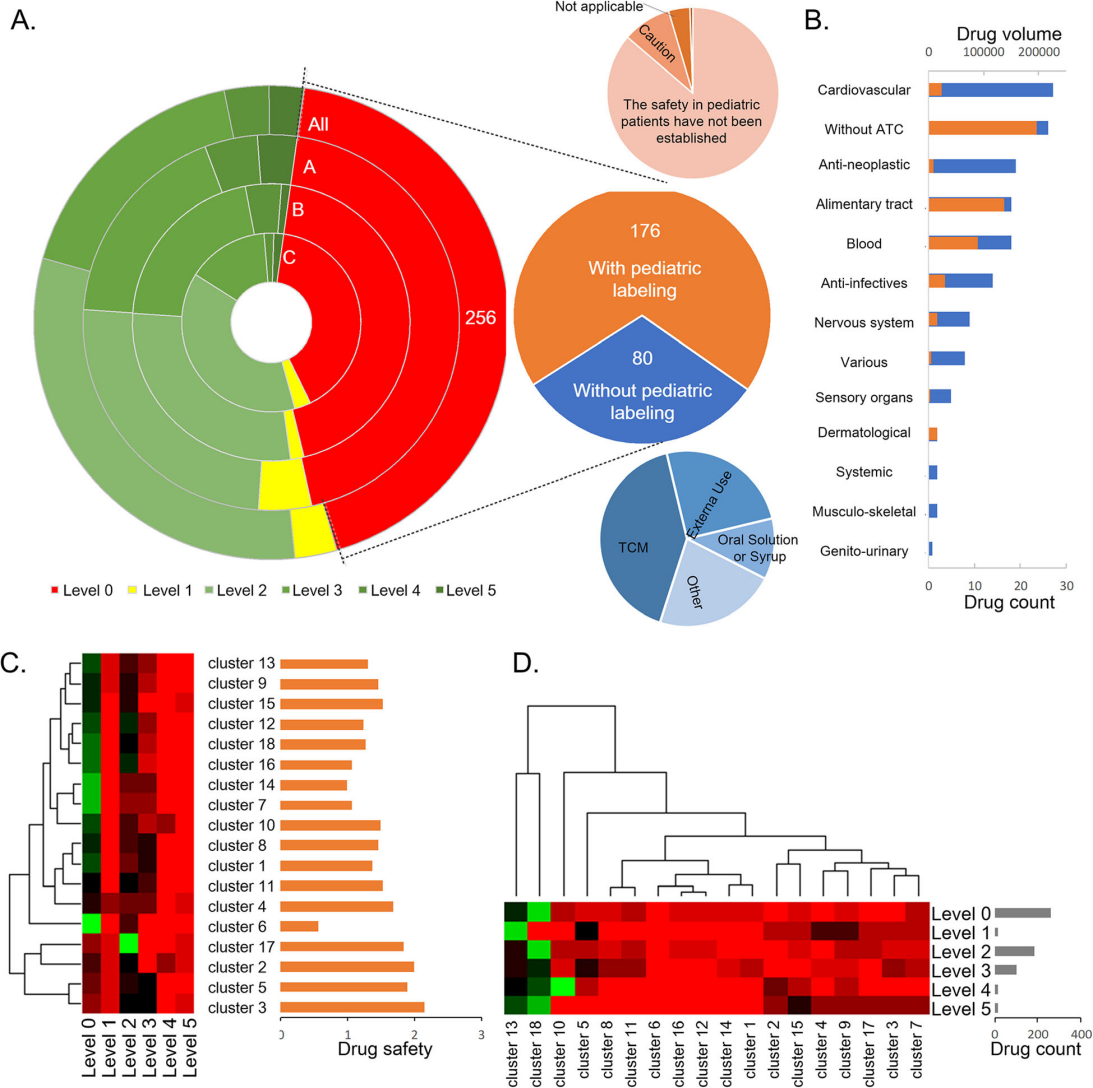
Drug safety in inpatient children. **a**. The doughnut of drug safety levels for all drugs used in inpatient children. The drug safety levels of groups (**a**, **b** and **c)** that were generated in Fig. 3a are also shown in the inner doughnuts. Drugs with a safety level of 0 were divided into two groups, and the details composition of the two groups are also shown in the two pie charts (top and bottom). **b**. A bar chart of the ATC first levels of the drugs used in pediatrics but with the pediatric labeling “the safety and efficacy in pediatric patients have not been established”. The number of drugs for each ATC classes is shown in blue bars. The volume of drug used is shown in the brown bars. **c**. The drug safety levels of the 18 drug clusters are plotted in the heatmap (green means larger proportion and red means smaller). The average drug safety level of each drug cluster is shown in the right bar chart. **d**. The distributions of drugs of different safety levels in the 18 drug clusters are plotted in the heatmap

All 591 drugs used in the children’s hospital were manually assigned a drug safety level, which was defined in the methods. As shown in Fig. 5a, 335 (56.68%) drugs had pediatric labeling and were applicable to children of certain ages based on their drug labels. However, there were still 256 (43.32%) safety level 0 drugs, which contributed to approximately 38.3% of the drug use in the children’s hospital. Among the safety level 0 drugs, there are 80 drugs without pediatric labeling. The drugs assumed to be safe in children, such as TCM, externally used drugs, oral solutions and syrups, comprise the vast majority of drugs in this group. If excluding this group of drugs, the safety level 0 drugs contribute to approximately 26.4% of all drug use. A total of 23 (3.89%) drugs that included explicit cautions or that were not applicable in pediatric labeling contributed to approximately 1.7% of all drug use. There are 153 (25.89%) drugs that have the following pediatric labeling but are still used in pediatrics: “the safety and efficacy in pediatric patients have not been established”. The bar chart shown in Fig. 5b lists the ATC first level classifications of these drugs. High-priority drugs that need to be studied in children are drugs for the cardiovascular system, which encompass 27 (4.57%) drugs. In addition, the most-used drugs in this list are drugs for the alimentary tract*.* From the doughnut in Fig. 5a, the percentage of drugs with safety levels above 3 in groups A and B is larger than the percentage in group C. Meanwhile, the percentage of drugs with a safety level of 2 in group C is relatively outstanding. These results also reflect that drugs in groups A and B are predominantly used in pediatric diseases.

The drug safety levels of 18 drug clusters are shown in Table 2 and plotted in Fig. 5c. Drug clusters 2, 3, 5 and 17 were separated from the other clusters due to their high drug safety level. These drug clusters are associated with neonatal diseases (cluster 2), diseases of the respiratory system (clusters 3 and 5), and epilepsy (cluster 17), respectively. Drug clusters 1, 4, 8 and 11 were grouped as having the second-highest drug safety level. These drug clusters are associated with infectious diseases (cluster 1), gastrointestinal diseases (cluster 4), general surgery problems (cluster 8), and ALL and its related infectious problems (cluster 11). Clusters 6 and 14 are two drug clusters with relatively low safety levels. Cluster 6 is a small cluster and contains only 7 drugs that were used to relieve symptoms such as pain, fever, nausea and vomiting during the treatment of ALL. Because there are 1 TCM and 2 externally used drugs without pediatric labeling in cluster 6, the drug safety level of this cluster was decreased. Cluster 14 was associated with drugs that were used during general surgery, especially for cerebral trauma, and more than half of these drugs were set to level 0. Another drug cluster with a lower safety level is cluster 16, which is associated with different cancers. The safety and efficacy of many antineoplastic agents, such as *Cisplatin* and *Carboplatin*, have not been well studied and established in children.

**Table 2:** Pediatric drug safety levels of drug clusters under different clinical conditions (details of the clinical conditions are shown in supplementary Table S1)

Cluster N.O.	Number of Drugs	Pediatric Drug Safety Level	
		Average	Standard Deviation
1	19	1.37	1.38
2	11	2.00	1.73
3	19	2.16	1.26
4	12	1.42	1.24
5	40	1.90	1.22
6	7	0.57	0.98
7	26	1.19	1.47
8	26	1.46	1.33
9	28	1.46	1.43
10	36	1.50	1.58
11	32	1.53	1.27
12	13	1.23	1.24
13	100	1.31	1.42
14	15	1.00	1.31
15	21	1.52	1.66
16	14	1.07	1.14
17	13	1.85	1.21
18	159	1.26	1.37
Total	591	1.41	1.39

The distribution of drugs of different safety levels in the drug clusters is shown in Fig. 5d. Excluding two special drug clusters (13 and 18), the drugs with a safety level of 5 were enriched in clusters 2, 3, 4, 7, 9, and 15. In addition to the drug clusters mentioned above, cluster 7 was associated with kidney diseases, and cluster 9 was associated with ALL and its subsequent convalescence. Cluster 15, which is associated with problems of preterm infant, is clearly at level 5. Drugs with a safety level of 4 with a hotspot at cluster 10 were associated with congenital heart disease. The average age of congenital heart disease patients treated in the children’s hospital was approximately 23 months, which required drugs with a safety level above 3. Cluster 5, which is associated with pneumonia, was also highlighted among drugs with safety levels of 3 and 1.

## Discussion

### Drug safety in children has improved but is still not optimistic

Half century ago, after the tragedy of fetal malformation from maternal ingestion of thalidomide, Dr. Harry Shirkey raised the concept of the “therapeutic orphan” and started the long journey of labeling all the therapeutic drugs for children [18]. In 1975, Wilson investigated the US Physicians’ Desk Reference and discovered that only 22% of drug labeling had adequate pediatric information [19]. In 2009, the proportion increased to 41% in the US [20]. The same progression also occurred in China. In 2007, there was a survey in the same children’s hospital investigated in this study that showed that 58% of the drugs used in the hospital had inadequate pediatric data [21]. Today, the number has clearly declined, and only 43% of drugs have been assigned to a safety level of 0. There has been impressive progress since that time. However, there are still many drugs that have not been approved for safety when used in children.

Before this study, we did not know how to measure the drug safety level under different pediatric clinical conditions, let alone how to improve it. As the data in Fig. 5 show, the high-priority drugs that need to be studied in children in China are primarily those for treating cardiovascular disease, alimentary tract-related diseases, cancers and trauma. As improving the drug safety in pediatric is a complex and arduous project that relies on multiple forces such as government, industry and clinical researches, we published all the 18 pediatric drug clusters at http://kb4md.org:4000/peddrugcluster to help experts in this domain to recognize this problem and improve it.

### The meaning and limitations of this method

With this method, quantitative drug safety levels under certain clinical conditions in pediatric care can be evaluated and compared. If there are longitudinal data, improvements can also be measured. Organizations such as the NICHD can use this method to identify drug use problems and measure improvements.

In addition, different ways to define drug safety can be integrated into this approach when the research topic does not concern about the pediatric drug safety. The drug clusters mined from EMR data show both the disease prevalence and pattern of drug use information and can be used to evaluate real-world drug safety.

The limitations of this method should also be noted. Because significant drug-diagnosis associations were calculated based on the Bonferroni-adjusted hypergeometric *P*-value, low-incidence diseases and orphan drugs are not enriched. Therefore, the fact that there are thousands of unique and low-incidence pediatric diseases that do not have significant associated drugs does not mean that there are no drugs for these conditions.

In addition, different drug clusters may be generated in different cultures and regions where different drugs are used. Therefore, clusters cannot be directly compared among diverse cultures. However, in a country with similar drug administration policy and drug supplies, the drug-diagnosis matrix will be similar for a targeted population. The drug-diagnosis comparing study can be used to identify “pharmaceutical gaps” among different populations.

## Conclusions

A computational method based on accumulated clinical data was proposed to extract significant drug-diagnosis associations and allow us to compare different drug use patterns among populations and generate drug clusters under different clinical conditions. With the drug safety level defined by each drug label, quantitative drug safety levels under certain clinical conditions can be calculated and quantitatively compared. This method will help to identify pediatric drug safety problems and to quantitatively measure the improvements in pediatric drug safety.

## Supplementary information

**Additional file 1: Figure S1.** Comparing the disease spectrum between the children’s hospital and the general hospital. **Figure S2.** Drug use volume of each drug cluster.**Table S1.** The Kernel Diagnosis Codes Set of the 18 Drug Clusters.

## Data Availability

The datasets generated and analyzed during the current study are available at http://kb4md.org:4000/peddrugcluster.

## Abbreviations

ALL: Acute Lymphoblastic Leukemia
ATC: Anatomical Therapeutic Chemical
EMA: European Medicines Agency
EMRs: Electronic Medical Records
ICD-10: International Classification of Disease, 10th edition
NICHD: National Institute of Child Health and Human Development
TCM: Traditional Chinese Medicine
