# Supplementary material for: A computational method to quantitatively measure pediatric drug safety using electronic medical records
Source: BMC Med Res Methodol. 2020 Jan 14;20:9. doi: 10.1186/s12874-020-0902-x (PMC6961323; doi:10.1186/s12874-020-0902-x)
Supplement: Supplementary file 1 — Additional file 1: Figure S1. Comparing the disease spectrum between the children’s hospital and the general hospital. Figure S2. Drug use volume of each drug cluster.Table S1. The Kernel Diagnosis Codes Set of the 18 Drug Clusters. [file 12874_2020_902_MOESM1_ESM.docx]

Supplemental Material

**Supplemental Source Code 1**

load("Orders.RData") # loading the medicication administration records data
load("Diagnosis.RData") # loading the diagnosis data
uVisitId<-unique(Orders$VISIT_ID) # get the inpatient visit id list
OrDiag<-subset(Diagnosis,VisitId %in% uVisitId,select = c('ICD10','VisitId')) # get the corresponding diagnosis of inpatients
VisitId.dict <- as.data.table(OrDiag$VisitId)[, list(list(.I)), by = OrDiag$VisitId]
DRUG.dict <- as.data.table(Orders$ORDER_TEXT)[,list(list(.I)), by = Orders$ORDER_TEXT]
DRUG.VisitId <- lapply(DRUG.dict$V1, function(x) unique(Orders$VISIT_ID[x]))
names( DRUG.VisitId) <- DRUG.dict$Orders
DRUG.INDEX <- lapply(DRUG.VisitId, function(x) VisitId.dict[OrDiag %in% x, unlist(V1)])
names(DRUG.INDEX) <- DRUG.dict$Orders
uDiag<-unique(OrDiag$ICD10)
#create the drug-diagnosis frequency matrix
FREQ <- matrix(0, length(DRUG.INDEX), length(uDiag), dimnames = list(names(DRUG.INDEX), uDiag))
for (i in 1:length(DRUG.INDEX)) {
 INDICES <- DRUG.INDEX[[i]]
 COUNTS <- table(unname(unlist(OrDiag[INDICES, 'ICD10'])))[uDiag]
 FREQ[i,] <- COUNTS
}
FREQ[is.na(FREQ)] <- 0
# create the enhanced p-value drug-diagnsis matrix
ENR <- matrix(NA, nrow(FREQ), ncol(FREQ), dimnames = list(row.names(FREQ), colnames(FREQ)))
FREQ.row <- rowSums(FREQ)
FREQ.col <- colSums(FREQ)
FREQ.sum <- sum(FREQ)
for (i in 1:nrow(ENR)) {
 rowsum <- FREQ.row[i]
 for (j in 1:ncol(ENR)) {
 colsum <- FREQ.col[j]
 p.value <- phyper(FREQ[i,j]-1,colsum,FREQ.sum-colsum,rowsum,lower.tail=F)
 ENR[i,j] <- p.value
 }
}

ENR <- ENR * nrow(ENR) * ncol(ENR) # The Bonferroni correction
ENR <- ifelse(ENR > 0.05, 0, 1) # to binary bits
DIST <- dist(ENR,method='binary') # distance matrix
# cluster boot
cluster.jaccard <- list()
for (i in 10:100) {
 this.boot <- clusterboot(DIST, B=100, bootmethod='boot', clustermethod=kmeansCBI, krange=i, seed=15555)
 cluster.jaccard[[i]] <- this.boot$bootresult
}

jmeans <- unlist(lapply(cluster.jaccard[10:100],function(x) mean(as.numeric(x))))
jse <- unlist(lapply(cluster.jaccard[10:100],function(x) mean(as.numeric(x))/sqrt(length(as.numeric(x)))))
errbar(x=10:50, y=jmelans, yplus=jmeans+jse, yminus=jmeans-jse, xlab='k (Number of Clusters)', ylab='Mean Jaccard Index (+/- SE)')
#the best results
best.boot<-clusterboot(DIST,B=100,bootmethod = 'boot',clustermethod = kmeansCBI,krange=36,seed = 15555)


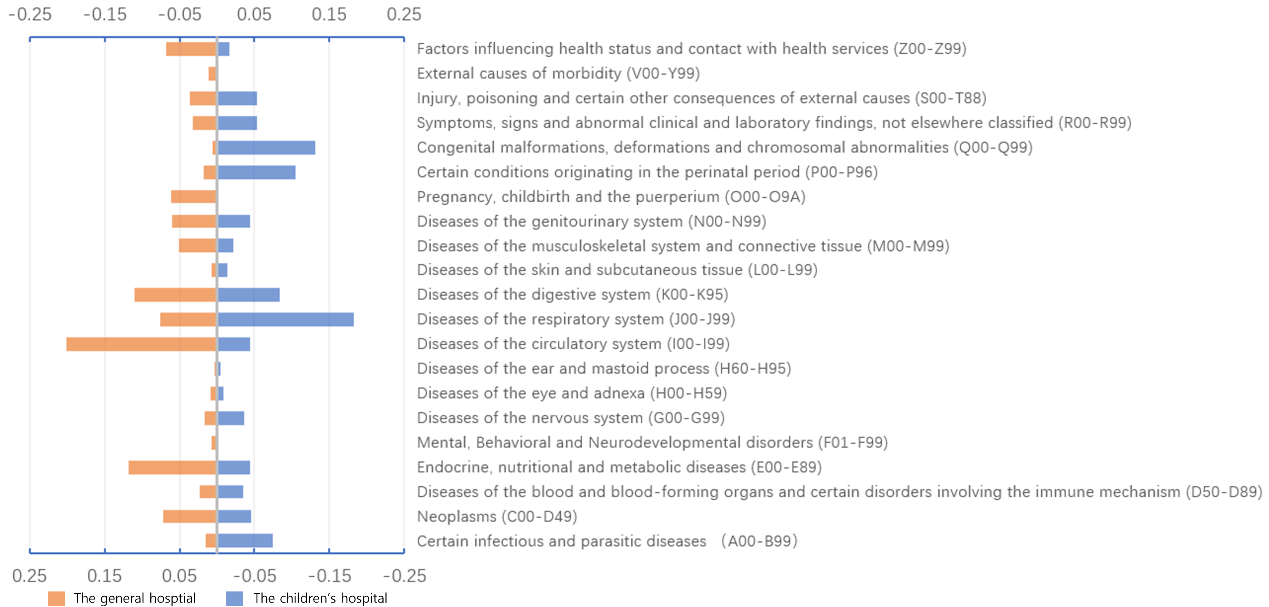


**Figure S1 Comparing the disease spectrum between the children’s hospital and the general hospital. The disease incidence of different type of diseases (based on the ICD-10 top category) were normalized and compared respectively.**


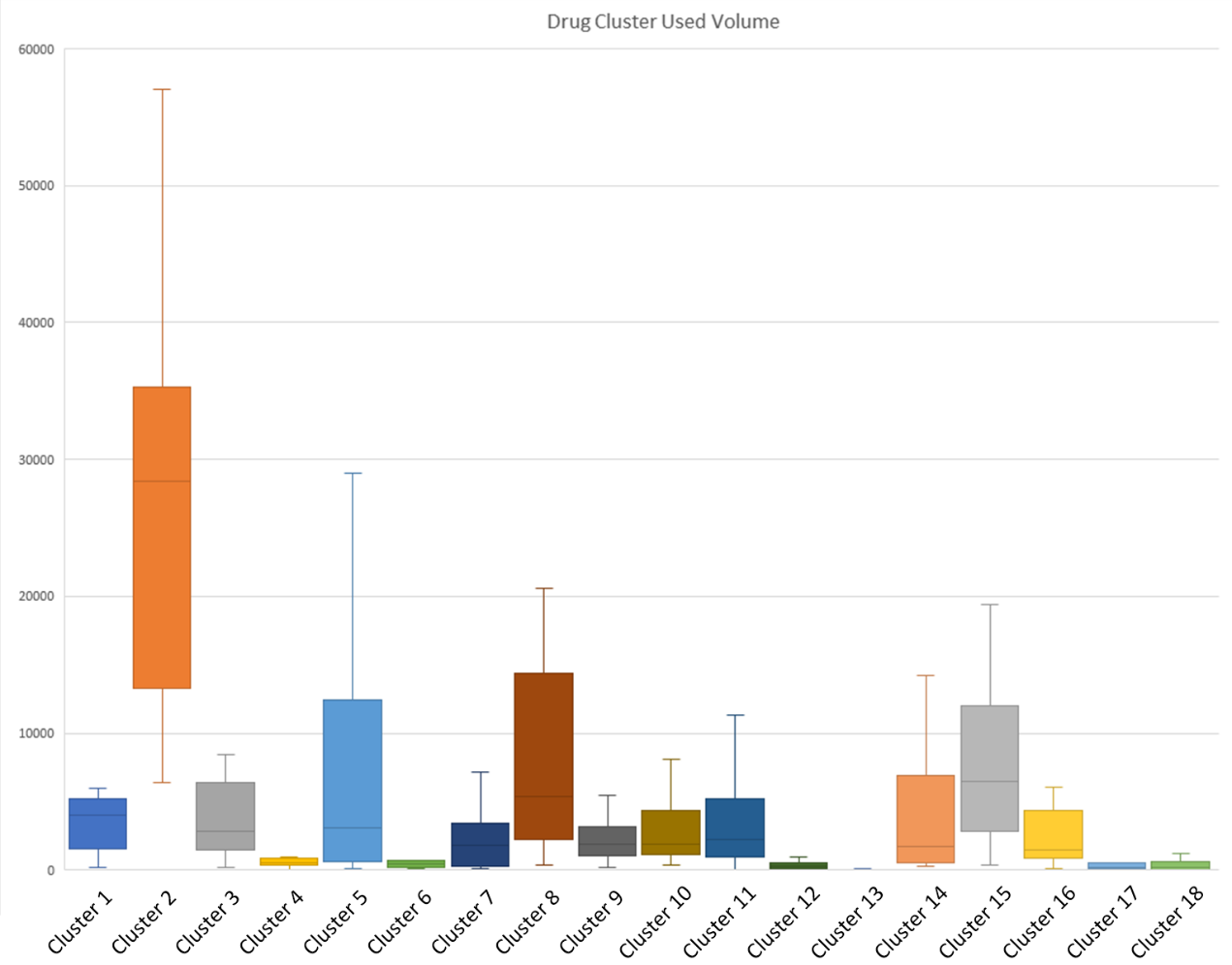


**Figure S2 Drug use volume of each drug cluster.**

Table S1 The Kernel Diagnosis Codes Set of the 18 Drug Clusters

| **Cluster  N.O.** | **Number of drugs** | **Kernel Diagnosis Codes Set** |
| --- | --- | --- |
| 1 | 19 | J06.900 Acute upper respiratory infection, unspecified  R56.001 Simple febrile convulsions  A86.x00 Unspecified viral encephalitis  J20.900 Acute bronchitis, unspecified  J03.901 Acute tonsillitis, unspecified  A41.900 Sepsis, unspecified organism  B08.501 Enteroviral vesicular pharyngitis |
| 2 | 11 | P10.901 Unspecified intracranial laceration and hemorrhage due to birth injury  P70.400 Other neonatal hypoglycemia  P07.101 Other low birth weight newborn  P36.901 Bacterial sepsis of newborn, unspecified  P05.100 Newborn small for gestational age  G93.002 Cerebral cysts  I62.900 Nontraumatic intracranial hemorrhage, unspecified  S00.004 Superficial injury of scalp  P07.300 Preterm [premature] newborn  P61.400 Other congenital anemias, not elsewhere classified  P55.100 ABO isoimmunization of newborn  P21.900 Asphyxia neonatorum  P59.901 Neonatal jaundice, unspecified |
| 3 | 19 | J35.100 Hypertrophy of tonsils  J35.200 Hypertrophy of adenoids  H65.400 Other chronic nonsuppurative otitis media  R06.501 Mouth breathing  J35.000 Chronic tonsillitis and adenoiditis  G47.301 Sleep apnea  J35.300 Hypertrophy of tonsils with hypertrophy of adenoids  J32.900 Chronic sinusitis, unspecified |
| 4 | 12 | K29.300 Chronic superficial gastritis  K29.801 Duodenitis  K20.x00 Esophagitis |
| 5 | 40 | J18.000 Bronchopneumonia, unspecified organism  I50.906 Myocardial injury  J18.902 Pneumonia  J45.903 Other and unspecified asthma |
| 6 | 7 | C91.000 Acute lymphoblastic leukemia |
| 7 | 26 | J06.900 Acute upper respiratory infection, unspecified  N04.900 Nephrotic syndrome  D69.004 Henoch’s purpura  D69.005+ Henoch-Schönlein purpura  N02.801 Iga nephropathy |
| 8 | 26 | N47.x01 Adherent prepuce  K35.900 Acute appendicitis  K37.x00 Unspecified appendicitis  N47.x00 Phimosis  K35.001 Acute appendicitis with localized peritonitis  Q55.606 Hidden penis  N36.000 Urethral fistula  K66.002 Peritoneal adhesions  Q54.900 Hypospadias  K65.900 Peritonitis  K35.901 Acute appendicitis with generalized peritonitis  Q55.604 Curvature of penis  K35.101 Appendiceal abscess |
| 9 | 28 | A41.900 Sepsis, unspecified organism  C91.000 Acute lymphoblastic leukemia  B44.102+ Aspergillus pneumonia  J18.900 Pneumonia  D61.903 Pancytopenia  Z54.900 Convalescence following unspecified treatment  A41.901 Septicopyemia  C92.500 Acute myelomonocytic leukemia |
| 10 | 36 | Q21.000 Ventricular septal defect  Q21.100 Atrial septal defect  Q24.900 Congenital malformation of heart, unspecified  I27.000 Primary pulmonary hypertension  Q25.000 Patent ductus arteriosus  I07.800 Other tricuspid valve diseases  I27.003 Severe pulmonary hypertension  I34.001 Mitral regurgitation  Q21.101 ASD, foramen ovale  Q21.300 Tetralogy of Fallot |
| 11 | 32 | C91.000 Acute lymphoblastic leukemia  A41.900 Sepsis, unspecified organism  B44.102+ Aspergillus pneumonia |
| 12 | 13 | N18.900 Chronic kidney disease, unspecified  D64.904 Secondary anemia  N18.001 Chronic kidney disease, stage 5  T86.001 Graft-versus-host reaction or disease  D61.900 Aplastic anaemia, unspecified  N04.900 Nephrotic syndrome  M32.900 Systemic lupus erythematosus, unspecified |
| 13 | 100 | NULL |
| 14 | 15 | S06.202 Cerebral contusion  S02.900 Fracture of skull  I62.101 Nontraumatic extradural haemorrhage  S00.004 Superficial injury of scalp  S09.900 Unspecified injury of head  G91.900 Hydrocephalus, unspecified  S06.804 Traumatic intracranial haemorrhage  S06.600 Traumatic subarachnoid haemorrhage  R90.000 Intracranial space-occupying lesion  Q28.200 Arteriovenous malformation of cerebral vessels  I62.000 Subdural haemorrhage  S27.301 Lung contusion |
| 15 | 21 | P07.300 Preterm [premature] newborn  P07.000 Extremely low birth weight  P22.000 Respiratory distress syndrome of newborn  Z38.500 Twin, unspecified as to place of birth  P07.101 Other low birth weight newborn  Q25.000 Patent ductus arteriosus  P77.x00 Necrotizing enterocolitis of newborn  P21.900 Birth asphyxia, unspecified  P27.100 Bronchopulmonary dysplasia originating in the perinatal period  P70.400 Other neonatal hypoglycaemia |
| 16 | 14 | D41.001 Neoplasm of kidney  C74.900 Malignant neoplasm of adrenal gland  C49.900 Malignant neoplasm of other connective and soft tissue  D48.900 Neoplasm of uncertain or unknown behaviour, unspecified  C64.x00 Malignant neoplasm of kidney  C91.000 Acute lymphoblastic leukemia  C83.700 Burkitt lymphoma  B44.102+ Aspergillus pneumonia  C22.200 Hepatoblastoma  C85.900 Non-Hodgkin lymphoma, unspecified |
| 17 | 13 | G40.901 Epilepsy, unspecified  G41.900 Status epilepticus, unspecified |
| 18 | 159 | I49.900 Cardiac arrhythmia, unspecified  E10.900 Type I diabetes mellitus  H26.900 Cataract, unspecified  T30.000 Burn of unspecified body region, unspecified degree  H52.701 Disorder of refraction, unspecified  E14.900 Diabetes NOS  P36.901 Bacterial sepsis of newborn, unspecified  K92.210 Gastrointestinal haemorrhage, unspecified  M08.900 Juvenile arthritis, unspecified  K50.900 Crohn disease, unspecified |

Table S2. The drugs and their safety level in each drug clusters.

| cluster | drug_CN | drug_EN | safety level |
| --- | --- | --- | --- |
| 1 | 利可君片 | Leucogen Tablets | 0 |
| 1 | 利巴韦林注射液 | Ribavirin Injection | 2 |
| 1 | 利巴韦林颗粒 | Ribavirin Granules | 0 |
| 1 | 地西泮片 | Diazepam Tablets | 3 |
| 1 | 奥拉西坦注射液 | Oxiracetam Injection | 0 |
| 1 | 对乙酰氨基酚口服溶液 | Paracetamol Oral Solution | 2 |
| 1 | 对乙酰氨基酚混悬滴剂 | Paracetamol Suspension Drop | 3 |
| 1 | 小儿伪麻美芬滴剂 | Pediatric Pseudoephedrine Hydrochloride and Dextrometharphan Hydrobromide Drops | 3 |
| 1 | 小儿柴桂退热颗粒 | Xiaoer Caigui Tuire Granules | 0 |
| 1 | 布洛芬混悬液 | Ibuprofen Suspension | 3 |
| 1 | 开喉剑喷雾剂 | Kaihoujian Spray | 0 |
| 1 | 愈酚甲麻那敏糖浆 | Guaifenesin，Methylephedrine Hydrochloride and Chlorphenamine Maleate Syrup | 0 |
| 1 | 水合氯醛糖浆 | Chloral Hydrate Syrup | 0 |
| 1 | 注射用头孢曲松钠 | Ceftriaxone Sodium for Injection | 3 |
| 1 | 注射用阿昔洛韦 | Aciclovir for Injection | 2 |
| 1 | 炉甘石洗剂 | Calamine Lotion | 0 |
| 1 | 盐酸伐昔洛韦分散片 | Valacyclovir Hydrochloride Dispersible Tablets | 2 |
| 1 | 磷酸奥司他韦胶囊 | Oseltamivir Phosphate Capsules | 3 |
| 1 | 蒲地蓝消炎口服液 | Phantiphlogistic oral solution | 0 |
| 2 | 50％葡萄糖注射液 | 50% Glucose Injection | 2 |
| 2 | 5％碳酸氢钠注射液 | 5% Sodium Bicarbonate Injection | 2 |
| 2 | 呋喃西林氧化锌搽剂 | furacilin and zinc oxide liniment | 0 |
| 2 | 开塞露 | Enema Glycerini | 2 |
| 2 | 氧氟沙星滴眼液 | Ofloxacin Eye Drops | 4 |
| 2 | 注射用头孢噻肟钠 | cefotaxime sodium injection | 4 |
| 2 | 注射用苯巴比妥钠 | Phenobarbital Sodium for Injection | 2 |
| 2 | 维生素K1注射液 | Vitamin K1 Injection | 5 |
| 2 | 聚维酮碘溶液 | Povidone Iodine Solution | 0 |
| 2 | 苯扎氯铵贴 | Benzalkonium Chloride Patches | 0 |
| 2 | 过氧化氢溶液 | Hydrogen Peroxide Solution | 1 |
| 3 | 0.9％氯化钠注射液 | 0.9% Sodium Chloride Injection | 2 |
| 3 | 丙酸氟替卡松鼻喷雾剂 | Fluticasone Propionate Nasal Spray | 1 |
| 3 | 呋麻滴鼻液 | Ephedrine Hydrochloride and Nitrofurazone Nasal Drops | 2 |
| 3 | 头孢克洛干混悬剂 | Cefaclor for Oral Suspension | 3 |
| 3 | 头孢克肟颗粒 | Cefixime Granules | 3 |
| 3 | 头孢羟氨苄片 | Cefadroxil Tablets | 2 |
| 3 | 康复新液 | Kangfuxin Solution | 0 |
| 3 | 注射用头孢呋辛钠 | Cefuroxime Sodium for Injection | 3 |
| 3 | 注射用矛头蝮蛇血凝酶 | Hemocoagulase Bothrops Atrox for Injection | 0 |
| 3 | 注射用血凝酶 | Hemocoagulase Atrox for Injection | 2 |
| 3 | 注射用阿莫西林钠克拉维酸钾 | Amoxicillin Sodium and Clavulanate Potassium for Injection | 5 |
| 3 | 盐酸左氧氟沙星滴耳液 | Levofloxacin Hydrochloride Ear Drops | 0 |
| 3 | 盐酸氨溴索口服溶液 | Ambroxol Hydrochloride Oral Solution | 3 |
| 3 | 盐酸氨溴索葡萄糖注射液 | Ambroxol Hydrochloride and Glucose Injection | 3 |
| 3 | 盐酸羟甲唑啉喷雾剂 | Oxymetazoline Hydrochloride Spray | 2 |
| 3 | 糠酸莫米松鼻喷雾剂 | Mometasone Furoate Nasal Spray | 2 |
| 3 | 葡萄糖氯化钠注射液 | Glucose and Sodium Chloride Injection | 2 |
| 3 | 酚麻美敏混悬液 | Paracetamol Pseudoephedrine Hydrochloride Dextromethorphan Hydrobromide and Chlorphenamine Maleate Suspension | 3 |
| 3 | 阿莫西林克拉维酸钾颗粒 | Amoxicillin and Clavulanate Potassium Granules | 3 |
| 4 | 克拉霉素片 | Clarithromycin Tablets | 3 |
| 4 | 多潘立酮片 | Domperidone Tablets | 1 |
| 4 | 奥美拉唑镁肠溶片 | Omeprazole Magnesium Entericcoated Tablets | 0 |
| 4 | 枫蓼肠胃康颗粒 | Maple Polygonum Chang Wei Kang Granules | 0 |
| 4 | 盐酸消旋山莨菪碱注射液 | Raceanisodamine Hydrochloride Injection | 2 |
| 4 | 胶体果胶铋胶囊 | Colloidal Bismuth Pectin Capsules | 0 |
| 4 | 西甲硅油乳剂 | Simethicone Emulsion | 3 |
| 4 | 酚酞含片 | Phenolphthalein Buccal Tablets | 2 |
| 4 | 钆双胺注射液 | Gadodiamide Injection | 3 |
| 4 | 钆喷酸葡胺注射液 | Gadopentetic acid Dimeglumine Salt Injection | 2 |
| 4 | 铝碳酸镁片 | Hydrotalcite Tablets | 0 |
| 4 | 阿莫西林克拉维酸钾片 | Amoxicillin and Clavulanate Potassium Tablets | 1 |
| 5 | 丙酸氟替卡松吸入气雾剂 | Fluticasone Propionate Inhaled Aerosol | 2 |
| 5 | 克拉霉素干混悬剂 | Clarithromycin for Suspension | 2 |
| 5 | 卡介菌纯蛋白衍生物 | Purified Protein Derivative of BCG(BCG-PPD) | 3 |
| 5 | 双歧杆菌三联活菌散 | Live Combined Bifidobacterium | 3 |
| 5 | 口服乳杆菌LB散 | Lactobacillus LB sachet | 2 |
| 5 | 口服补液盐散(Ⅰ) | Oral Rehydration Salts Powder(I) | 0 |
| 5 | 吸入用布地奈德混悬液 | Budesonide Suspension for Inhalation | 3 |
| 5 | 吸入用异丙托溴铵溶液 | Ipratropium Bromide Solution for Inhalation | 1 |
| 5 | 呋麻滴鼻液（Ⅱ） | Ephedirne Hydrichloride and Nitrofurazone Nasal Drops (II) | 0 |
| 5 | 头孢地尼分散片 | Cefdinir Dispersible Tablets | 3 |
| 5 | 婴儿健脾口服液 | Infant Jianpi oral liquid | 3 |
| 5 | 孟鲁司特钠咀嚼片 | Montelukast Sodium Chewable Tablets | 3 |
| 5 | 富马酸酮替芬片 | Ketotifen Fumarate Tablets | 2 |
| 5 | 小儿咳喘灵口服液 | Pediatric KeChuanLing Oral Liquid | 3 |
| 5 | 小儿消积止咳口服液 | Pediatric antitussive oral liquid | 3 |
| 5 | 布地奈德福莫特罗粉吸入剂 | Budesonide and Formoterol Fumarate Powder for Inhalation | 1 |
| 5 | 布拉氏酵母菌散 | Saccharomyces boulardii sachets | 3 |
| 5 | 枯草杆菌二联活菌颗粒 | Combined Bacillus Subtilis and Enterococcus Faecium Granules with Multivitamines | 4 |
| 5 | 氨溴特罗口服溶液 | Ambroxol Hydrochloride and Clenbuterol Hydrochloride Oral Solution | 2 |
| 5 | 水合氯醛灌肠液 | Chloral Hydrate Enemas | 0 |
| 5 | 水合氯醛粉 | Chloral Hydrate Powder | 0 |
| 5 | 沙美特罗替卡松粉吸入剂 | Salmeterol Xinafoateand Fluticasone Propionate Powder for Inhalation | 2 |
| 5 | 注射用乳糖酸红霉素 | Erythromycin Lactobionate for Injection | 0 |
| 5 | 注射用乳糖酸阿奇霉素 | Azithromycin Lactobionate for Injection | 0 |
| 5 | 注射用甲泼尼龙琥珀酸钠 | Methylprednisolone Sodium Succinate for Injection | 3 |
| 5 | 注射用阿奇霉素 | Azithromycin for Injection | 3 |
| 5 | 注射用阿莫西林钠舒巴坦钠 | Amoxicillin Sodium and Sulbactam sodium for Injection | 3 |
| 5 | 灭菌注射用水 | Sterile Water for Injection | 0 |
| 5 | 生理氯化钠溶液 | Sodium Chloride Physiological Solution | 0 |
| 5 | 盐酸丙卡特罗口服溶液 | Procaterol Hydrochloride Oral Solution | 3 |
| 5 | 盐酸丙卡特罗片 | Procaterol Hydrochloride Tablets | 2 |
| 5 | 盐酸氨溴索注射液 | Ambroxol Hydrochloride Injection | 3 |
| 5 | 盐酸西替利嗪滴剂 | Cetirizine Dihydrochloride Oral Drops | 2 |
| 5 | 硫酸沙丁胺醇气雾剂 | Salbutamol Sulphate Aerosol | 2 |
| 5 | 硫酸特布他林雾化液 | Terbutaline Sulphate Solution for Nebulization | 2 |
| 5 | 蒙脱石散 | Montmorillonite Powder | 3 |
| 5 | 重组人干扰素α1b滴眼液 | Recombinant Human Interferon α2b Eye Drops | 2 |
| 5 | 阿奇霉素干混悬剂 | Azithromycin for Suspension | 2 |
| 5 | 阿奇霉素片 | Azithromycin Tablets | 1 |
| 5 | 鼻渊通窍颗粒 | BiYuanTongQiao Granule | 0 |
| 6 | 双氯芬酸二乙胺乳胶剂 | Diclofenac Diethylamine Emulgel | 0 |
| 6 | 注射用门冬酰胺酶 | Asparaginase for lnjection | 0 |
| 6 | 玉屏风口服液 | Yupingfeng Oral Liquid | 0 |
| 6 | 盐酸昂丹司琼片 | Ondansetron Hydrochloride Tablets | 0 |
| 6 | 硫酸镁粉 | Magnesium Sulrate | 0 |
| 6 | 美敏伪麻溶液 | Pseudoephedrine Hydrochloride Chlorphenamine Maleate and Dextromethorphan Hydrobromide Solution | 2 |
| 6 | 重组人表皮生长因子凝胶 | Recombinant Human Epidermal Growth Factor Gel | 2 |
| 7 | 他克莫司胶囊 | Tacrolimus Capsules | 2 |
| 7 | 双嘧达莫片 | Dipyridamole Tablets | 1 |
| 7 | 右旋糖酐40葡萄糖注射液 | Dextran 40 Glucose Injection | 0 |
| 7 | 吗替麦考酚酯分散片 | Mycophenolate Mofetil Dispersible Tablets | 0 |
| 7 | 吗替麦考酚酯胶囊 | Mycophenolate Mofetil Capsules | 2 |
| 7 | 小儿热速清颗粒 | Infant Reshuqing Granule | 3 |
| 7 | 愈酚伪麻口服溶液 | Compound Guaifenesin and Pseudoephedrine Hydrochloride Oral Solution | 3 |
| 7 | 施保利通片 | Esberitox Tablets | 0 |
| 7 | 来氟米特片 | Leflunomide Tablets | 0 |
| 7 | 氯雷他定糖浆 | Loratadine Syrup | 2 |
| 7 | 注射用氨苄西林钠舒巴坦钠 | Ampicillin Sodium and Sulbactam Sodium for lnjection | 5 |
| 7 | 甲泼尼龙片 | Methylprednisolone Tablets | 3 |
| 7 | 白芍总苷胶囊 | Total Glucosides of White Paeony Capsules | 0 |
| 7 | 盐酸左旋咪唑片 | Levamisole Hydrochloride Tablets | 0 |
| 7 | 盐酸西替利嗪糖浆 | Cetirizine Hydrochloride Syrup | 2 |
| 7 | 碳酸钙D3咀嚼片(Ⅱ) | Calcium Carbonate and Vitamin D3 Chewable Tablets(II) | 0 |
| 7 | 维生素D滴剂 | Vitamin D Drops | 2 |
| 7 | 西咪替丁注射液 | Cimetidine Injection | 0 |
| 7 | 那屈肝素钙注射液 | Nadroparin Calcium Injection | 0 |
| 7 | 醋酸泼尼松片 | Prednisolone Acetate Tablets | 3 |
| 7 | 醋酸钙颗粒 | Calcium Acetate Granules | 0 |
| 7 | 金莲清热泡腾片 | JinLianQingRe Effervescent Tablets | 3 |
| 7 | 阿魏酸哌嗪片 | Piperazine Ferulate Tablets | 0 |
| 7 | 马来酸依那普利片 | Enalapril Maleate Tablets | 0 |
| 7 | 高锰酸钾外用片 | Potassium Permanganate for External Use | 0 |
| 7 | 黄芪片 | HuangQi Tablets | 0 |
| 8 | 10％氯化钾注射液 | 10% Potassium Chloride Injection | 0 |
| 8 | 5％葡萄糖氯化钠注射液 | 5% Glucose and Sodium Chloride Injection | 0 |
| 8 | 吲哚美辛栓 | Indometacin Suppositories | 2 |
| 8 | 喷他佐辛注射液 | Pentazocine Injection | 2 |
| 8 | 复方利多卡因乳膏 | Compound Lidocaine Cream | 3 |
| 8 | 头孢克肟胶囊 | Cefixime Capsules | 3 |
| 8 | 小儿复方氨基酸注射液 | Pediatric Compound Amino Acid Injection | 0 |
| 8 | 小麦纤维素颗粒 | Testa Triticum Tricum Purif | 3 |
| 8 | 恒古骨伤愈合剂 | OSTEOKING | 2 |
| 8 | 氨基酸注射液 | Amino Acid Injection | 2 |
| 8 | 注射用哌拉西林钠他唑巴坦钠 | Piperacillin Sodium and Tazobactam Sodium for Injection | 3 |
| 8 | 注射用复合辅酶 | Coenzyme Complex for Injection | 0 |
| 8 | 注射用头孢地嗪钠 | Cefodizime Sodium for Injection | 0 |
| 8 | 注射用奥硝唑 | Ornidazole for Injection | 2 |
| 8 | 注射用奥美拉唑钠 | Omeprazole Sodium for Injection | 0 |
| 8 | 注射用尖吻蝮蛇血凝酶 | Haemocoagulase Agkistrodon for Injection | 0 |
| 8 | 注射用帕尼培南倍他米隆 | Panipenem and Betamipron for Injection | 3 |
| 8 | 注射用甲硝唑磷酸二钠 | Metronidazole Disodium Phosphate for Injection | 2 |
| 8 | 注射用白眉蛇毒血凝酶 | Hemocoagulase for Injection | 2 |
| 8 | 注射用盐酸头孢替安 | Cefotiam Hydrochloride for Injection | 3 |
| 8 | 注射用盐酸头孢甲肟 | Metronidazole Disodium Phosphate for Injection | 3 |
| 8 | 注射用脂溶性维生素Ⅰ | Fat-soluble Vitamin For Injection (I) | 3 |
| 8 | 热淋清颗粒 | relinqing granules | 0 |
| 8 | 盐酸奥布卡因凝胶 | Oxybuprocaine Hydrochloride Gel | 0 |
| 8 | 红霉素软膏 | Erythromycin Ointment | 0 |
| 8 | 苯扎氯铵溶液 | Benzalkonium Chloride Solution | 0 |
| 9 | L-谷氨酰胺胍仑酸钠颗粒 | L-Glutamine and Sodium Gualenate Granules | 0 |
| 9 | 伏立康唑片 | Voriconazole Tablets | 2 |
| 9 | 利奈唑胺注射液 | Linezolid Injection | 5 |
| 9 | 利奈唑胺片 | Linezolid Tablets | 4 |
| 9 | 制霉素搽剂 | Mycophenolate liniment | 0 |
| 9 | 制霉素甘油 | Mycophenolate glycerol | 0 |
| 9 | 培门冬酶注射液 | Pegaspargase Injection | 2 |
| 9 | 复方氯己定含漱液 | Compound Chlorhexidine Gargle | 0 |
| 9 | 复方磺胺甲噁唑片 | Compound Sulfamethoxazole Tablets | 3 |
| 9 | 多磺酸粘多糖乳膏 | Mucopolysaccharide Polysulfate Cream | 0 |
| 9 | 头孢地尼胶囊 | Cefdinir Capsules | 3 |
| 9 | 注射用亚叶酸钙 | Calcium Folinate for Injection | 0 |
| 9 | 注射用伏立康唑 | Voriconazole for Injection | 1 |
| 9 | 注射用右丙亚胺 | Dexrazoxane for Injection | 0 |
| 9 | 注射用甲氨蝶呤 | Methotrexate for Injection | 2 |
| 9 | 注射用盐酸伊达比星 | Idarubicin Hydrochloride for Injection | 2 |
| 9 | 注射用盐酸表柔比星 | Epirubicin Hydrochloride for Injection | 2 |
| 9 | 注射用醋酸卡泊芬净 | Caspofungin Acetate for Injection | 3 |
| 9 | 注射用重组人白介素-11 | Recombinant Human Interleukin-11 for Injection | 0 |
| 9 | 注射用阿糖胞苷 | Cytarabine for Injection | 2 |
| 9 | 甲钴胺片 | Mecobalamin Tablets | 0 |
| 9 | 百蕊颗粒 | Bairui Oral Granules | 0 |
| 9 | 盐酸异丙嗪片 | Promethazine Hydrochloride Tablets | 3 |
| 9 | 碳酸氢钠片 | Sodium Bicarbonate Tablets | 1 |
| 9 | 肺力咳合剂 | Feilike Mixture | 2 |
| 9 | 芙朴感冒颗粒 | Fu Pu cold granules | 0 |
| 9 | 醋酸地塞米松片 | Dexamethasone Acetate Tablets | 2 |
| 9 | 重组人粒细胞刺激因子注射液 | Recombinant Human Granulocyte Colony-stimulating Factor Injection | 2 |
| 10 | 1％盐酸普鲁卡因注射液 | 1% Procaine Hydrochloride Injection | 0 |
| 10 | 20％人血白蛋白注射液 | 20% Human Albumin Injection | 0 |
| 10 | 5％氯化钙注射液 | 5% Calcium Chloride Injection | 0 |
| 10 | 丙泊酚注射液 | Propofol Injection | 2 |
| 10 | 乳酸米力农注射液 | Milrinone Lactate Injection | 2 |
| 10 | 人凝血酶原复合物 | Human Prothrombin Complex | 0 |
| 10 | 前列地尔注射液 | Alprostadil Injection | 3 |
| 10 | 卡托普利片 | Captopril Tablets | 3 |
| 10 | 吲哚美辛肠溶片 | Indometacin Enteric-coated Tablets | 2 |
| 10 | 呋塞米注射液 | Furosemide Injection | 4 |
| 10 | 呋塞米片 | Furosemide Tablets | 4 |
| 10 | 地高辛口服溶液 | Digoxin Oral Solution | 4 |
| 10 | 左卡尼汀注射液 | Levocarnitine Injection | 2 |
| 10 | 果糖二磷酸钠口服液 | Fructose　Sodium　Diphosphate　Oral　Solution | 0 |
| 10 | 枸橼酸芬太尼注射液 | Sufentanil Citrate Injection | 0 |
| 10 | 枸橼酸西地那非片 | Sildenafil Citrate Tablets | 0 |
| 10 | 氢氯噻嗪片 | Hydrochlorothiazide Tablets | 4 |
| 10 | 氨甲环酸注射液 | Tranexamic Acid Injection | 0 |
| 10 | 注射用丙氨酰谷氨酰胺 | Alanyl Glutamine for Injection | 0 |
| 10 | 注射用头孢拉定 | Cefradine for Injection | 3 |
| 10 | 注射用甲磺酸酚妥拉明 | Phtneolamine Mesilate For Injection | 2 |
| 10 | 注射用磷酸肌酸钠 | Creatine Phosphate Sodium for Injection | 0 |
| 10 | 注射用维库溴铵 | Vecuronium Bromide for Injection | 4 |
| 10 | 注射用胸腺法新 | Thymosin Alpha-1 for Injection | 0 |
| 10 | 玻璃酸钠滴眼液 | Sodium Hyaluronate Eye Drops | 0 |
| 10 | 盐酸吗啡注射液 | Morphine Hydrochloride Injection | 2 |
| 10 | 盐酸多巴胺注射液 | Dopamine Hydrochloride Injection | 0 |
| 10 | 盐酸多巴酚丁胺注射液 | Dobutamine Hydrochloride Irljection | 0 |
| 10 | 盐酸异丙肾上腺素注射液 | Isoprenaline Hydrochloride Injection | 0 |
| 10 | 盐酸曲马多注射液 | TRAMADOL HYDROCHLORIDE INJECTION | 3 |
| 10 | 盐酸纳洛酮注射液 | Naloxone Hydrochloride Injection | 4 |
| 10 | 硫酸鱼精蛋白注射液 | Protamine Sulfate Injection | 2 |
| 10 | 米力农注射液 | Milrinone Injection | 0 |
| 10 | 胰岛素注射液 | Insulin Injection | 2 |
| 10 | 螺内酯片 | Spironolactone Tablet | 0 |
| 10 | 阿司匹林肠溶片 | Aspirin Entericcoated Tablets | 2 |
| 11 | 10％氯化钾口服溶液 | 10% Potassium Chloride Oral Solution | 2 |
| 11 | 乳果糖口服溶液 | Lactulose Oral Solution | 3 |
| 11 | 人纤维蛋白原 | Human Fibrinogen | 0 |
| 11 | 地塞米松磷酸钠注射液 | Dexamethasone Sodium Phosphate Injection | 2 |
| 11 | 复方甘草酸苷片 | Stronger Neo-Minophagen C | 2 |
| 11 | 复方福尔可定口服溶液 | Compound Pholcodine Oral Solution | 3 |
| 11 | 巯嘌呤片 | Azathioprine　Tablets | 2 |
| 11 | 氨甲苯酸注射液 | Aminomethylbenzoic Acid Injection | 0 |
| 11 | 氯化钾片 | Potassium Chloride Tablets | 0 |
| 11 | 氯化钾缓释片 | Sustained-Release Potassium Chloride Tablets | 0 |
| 11 | 氯雷他定片 | Loratadine Tablets | 3 |
| 11 | 注射用丁二磺酸腺苷蛋氨酸 | Ademetionine 1 4-Butanedisulfonate for Injection | 0 |
| 11 | 注射用亚胺培南西司他丁钠 | Imipenem and Cilastatin Sodium for Injection | 3 |
| 11 | 注射用头孢哌酮钠舒巴坦钠 | Cefoperazone Sodium and Sulbactam Sodium for Injection | 3 |
| 11 | 注射用盐酸米托蒽醌 | Mitoxantrone Hydrochloride for Injection | 0 |
| 11 | 注射用美罗培南 | Meropenem for Injection | 3 |
| 11 | 注射用还原型谷胱甘肽钠 | Reduced Glutathione for Injection | 0 |
| 11 | 注射用醋酸曲普瑞林 | Triptorelin Acetate for Injection | 2 |
| 11 | 注射用高三尖杉酯碱 | Homoharringtonine for Injection | 2 |
| 11 | 熊去氧胆酸胶囊 | Ursodeoxycholic Acid Capsules | 2 |
| 11 | 甲氨蝶呤注射液 | Methotrexate Injection | 3 |
| 11 | 甲氨蝶呤片 | Methotrexate Tablets | 0 |
| 11 | 盐酸利多卡因注射液 | Lidocaine Hydrochloride Injection | 3 |
| 11 | 盐酸格拉司琼注射液 | Granisetron Hydrochloride Injection | 0 |
| 11 | 盐酸甲氧氯普胺注射液 | Methyl Hydrochloride Injection | 2 |
| 11 | 碳酸钙D3咀嚼片 | Calcium Carbonate and Vitamin D3 Chewable Tablets | 2 |
| 11 | 肝素钠乳膏 | Heparin Sodiun Cream | 0 |
| 11 | 葡醛内酯片 | Glucurolactone Tablets | 2 |
| 11 | 辅酶Q10片 | Ubidecarenone Tablets | 0 |
| 11 | 酚磺乙胺注射液 | Etamsylate Injection | 3 |
| 11 | 重组人促红素注射液(CHO细胞) | Recombinant Human Erythropoietin Injection(CHO Cell) | 2 |
| 11 | 黄芪生脉饮 | HuangQiShengmai drink | 0 |
| 12 | 叶酸片 | Folic Acid Tablets | 2 |
| 12 | 注射用磷酸氟达拉滨 | Fludarabine Phosphate for Injection | 0 |
| 12 | 环孢素口服液 | Cyclosporine Oral Solution | 2 |
| 12 | 环孢素注射液 | Cyiclosporin Injection | 2 |
| 12 | 环孢素软胶囊 | Cyclosporin Soft Capsules | 2 |
| 12 | 硝苯地平片 | Nifedipine Tablets | 0 |
| 12 | 缬沙坦胶囊 | Trochisci Levamisole Hydrochloridi | 0 |
| 12 | 腹膜透析液(乳酸盐-G1.5%) | :Peritoneal Dialysis Solution(Lactate-G1.5%) | 0 |
| 12 | 腹膜透析液(乳酸盐-G2.5%) | :Peritoneal Dialysis Solution(Lactate-G2.5%) | 0 |
| 12 | 苯磺酸氨氯地平片 | Amlodipine Besylate Tablets | 2 |
| 12 | 葡萄糖酸亚铁糖浆 | Ferrous Cluconate Syru | 3 |
| 12 | 醋酸氯己定粉 | Compound Chlorhexidine Hydrochloride Dusting | 0 |
| 12 | 骨化三醇胶丸 | Calcitriol Soft Capsules | 3 |
| 13 | (幼)维生素AD滴剂 | (child) Vitamin A and D Drops | 3 |
| 13 | 2％盐酸普鲁卡因注射液 | 2% procaine hydrochloride injection | 0 |
| 13 | 乳酸钠林格注射液 | Sodium Lactate Ringer's Injection | 2 |
| 13 | 五维牛磺酸口服溶液 | Five Vitamins and Taurine Oral Solution | 2 |
| 13 | 伤科灵喷雾剂 | ShangKeLing Spray | 0 |
| 13 | 健儿清解液 | Jian'er Qing Jie Ye | 3 |
| 13 | 儿泻停颗粒 | ErXieTing Granules | 3 |
| 13 | 克霉唑乳膏 | Clotrimazole Cream | 0 |
| 13 | 兔抗人胸腺细胞免疫球蛋白 | Rabbit Anti-human Thymocyte Immunoglobulin | 0 |
| 13 | 八宝惊风散 | BaBaoJingFengSan | 3 |
| 13 | 利多卡因气雾剂 | Lidocaine Aerosol | 3 |
| 13 | 利巴韦林气雾剂 | Ribavirin Aerosol Suspension | 2 |
| 13 | 单唾液酸四己糖神经节苷脂钠注射液 | Monosialotetrahexosylganglioside Sodium Injection | 2 |
| 13 | 双歧杆菌三联活菌胶囊 | Bifid Triple Viable Capsules Dissolving at Intestines | 0 |
| 13 | 双黄连口服液 | Shuanghuanglian Oral Liquid | 2 |
| 13 | 右旋糖酐铁口服液 | Iron Dextran Oral Solution | 2 |
| 13 | 右旋糖酐铁口服溶液 | Iron Dextran Oral Solution | 3 |
| 13 | 吡拉西坦片 | Piracetam Tablets | 3 |
| 13 | 地奈德乳膏 | Desonide Cream | 2 |
| 13 | 地屈孕酮片 | Dydrogesterone Tablets | 0 |
| 13 | 地氯雷他定干混悬剂 | Desloratadine for Suspension | 3 |
| 13 | 复合维生素B片 | Compound Vitamin B Tablets | 2 |
| 13 | 复方愈创木酚磺酸钾口服溶液 | Compound Guaiacol Potassium Sulfonale oral Solution | 0 |
| 13 | 复方氢溴酸右美沙芬糖浆 | Compound Dextromethorphan Hydrobromide Syrup | 0 |
| 13 | 复方消化酶片 | Compound Digestive Enzyme Tablets | 2 |
| 13 | 复方消化酶胶囊 | Compound Digestive Enzyme Capsules | 0 |
| 13 | 复方鲜竹沥液 | Compound fresh bamboo juice | 0 |
| 13 | 大补阴丸 | large yin-nourishing pill | 0 |
| 13 | 天一止咳糖浆 | Tianyi cough Syrup | 0 |
| 13 | 头孢克洛胶囊 | Cefaclor Capsules | 3 |
| 13 | 妥布霉素滴眼液 | Tobramycin Eye Drops | 0 |
| 13 | 小儿惊风七厘散 | XiaoerJingFengQiLiSan | 3 |
| 13 | 小儿智力糖浆 | Child mental syrup | 2 |
| 13 | 小牛血去蛋白提取物眼用凝胶 | Deproteinized Calfblood Extract Eye Gel | 0 |
| 13 | 布地奈德粉吸入剂 | Budesonide Powder for Inhalation | 2 |
| 13 | 布地奈德鼻喷雾剂 | Budesonide Nasal Spray | 2 |
| 13 | 庆大霉素普鲁卡因维B12颗粒 | Gentamycin Sulfate Procaine Hydrochloride and Vitamin B12 Granules | 0 |
| 13 | 异烟肼注射液 | Isoniazid Injection | 4 |
| 13 | 异烟肼片 | Isoniazid Tablets | 2 |
| 13 | 更昔洛韦眼用凝胶 | Ganciclovir Ophthalmic Gel | 0 |
| 13 | 柴黄颗粒 | Chai Huang granule | 0 |
| 13 | 标准桃金娘油胶囊 | Myrtol Standardized Enteric Coated Soft Caosules | 0 |
| 13 | 气滞胃痛颗粒 | qizhi weitong granules | 0 |
| 13 | 氟马西尼注射液 | Flumazenil Injection | 0 |
| 13 | 氨甲苯酸片 | Aminomethylbenzoic Acid Tablets | 0 |
| 13 | 注射用两性霉素B脂质体 | Amphotericin B Liposome for Injection | 2 |
| 13 | 注射用头孢他啶 | Ceftazidime for Injection | 2 |
| 13 | 注射用头孢美唑钠 | Cefmetazole Sodium for Injection | 2 |
| 13 | 注射用替加环素 | Tigecycline for Injection | 0 |
| 13 | 注射用氟氧头孢钠 | Flomoxef Sodium for Injection | 5 |
| 13 | 注射用牛肺表面活性剂 | Calf Pulmonary Surfactant for Injection | 4 |
| 13 | 注射用盐酸头孢吡肟 | Cefepime Hydrochloride for Injection | 3 |
| 13 | 注射用盐酸平阳霉素 | Bleomycin A5 Hydrochloride for Injection | 0 |
| 13 | 注射用硝普钠 | Sodium Nitroprusside for Injection | 2 |
| 13 | 注射用硫代硫酸钠 | Sodium Thiosulfate for Injection | 0 |
| 13 | 注射用赖氨匹林 | Lysine Acetylsalicylate for Injection | 3 |
| 13 | 消旋山莨菪碱片 | Racanisodamine Tablets | 1 |
| 13 | 清咳平喘颗粒 | QingKePing Granules | 0 |
| 13 | 珍珠明目滴眼液 | ZhenZhuMingMu eye drops | 0 |
| 13 | 珍黄胶囊 | ZhenHuang Capsules | 0 |
| 13 | 甘霖洗剂 | Ganlin Xiji | 0 |
| 13 | 甲氧氯普胺片 | Metoclopramide Tablets | 2 |
| 13 | 甲紫溶液 | Methylrosanilinium Chloride Solution | 0 |
| 13 | 白消安注射液 | Busulfan Injection | 0 |
| 13 | 盐酸去氧肾上腺素注射液 | Phenylephrine Hydrochloride Injection | 0 |
| 13 | 盐酸哌替啶注射液 | Pethidine Hydrochloride Injection | 2 |
| 13 | 盐酸奥洛他定滴眼液 | Olopatadine Hydrochloride Eye Drops | 2 |
| 13 | 盐酸托莫西汀胶囊 | Atomoxetine Hydrochloride Capsules | 2 |
| 13 | 盐酸氮卓斯汀鼻喷剂 | Azelastine Hydrochloride Nasal Spray | 1 |
| 13 | 盐酸甲氯芬酯胶囊 | Meclofenoxate Hydrochloride Capsules | 2 |
| 13 | 盐酸索他洛尔片 | Sotalol Hydrochloride Tablets | 0 |
| 13 | 盐酸维拉帕米注射液 | Verapamil Hydrochloride Injection | 4 |
| 13 | 盐酸阿扎司琼注射液 | Azasetron Hydrochloride Injection | 0 |
| 13 | 眼氨肽滴眼液 | Ocular Extractives Eye Drops | 0 |
| 13 | 硝酸异山梨酯片 | Isosorbide Dinitrate Tablets | 0 |
| 13 | 硫酸妥布霉素注射液 | Tobramycin Sulfate Injection | 5 |
| 13 | 硫酸特布他林片 | Terbutaline Sulphate Tablets | 2 |
| 13 | 硫酸镁注射液 | Magnesium Sulfate Injection | 2 |
| 13 | 硫酸阿米卡星注射液 | Amikacin Sulfate Injection | 0 |
| 13 | 秋泻灵合剂 | QiuXieLingHeJi | 3 |
| 13 | 维生素A棕榈酸酯眼用凝胶 | Vitamin A Palmitate Eye Gel | 0 |
| 13 | 维生素B1注射液 | Vitamin B1 Injection | 0 |
| 13 | 维生素D3注射液 | Vitamin D3 Injection | 3 |
| 13 | 羚羊感冒口服液 | Antelope's cold oral liquid | 0 |
| 13 | 聚桂醇注射液 | Lauromacrogol Injection | 0 |
| 13 | 肠内营养粉剂（TP） | Enteral Nutritional Emulsion(TP) | 2 |
| 13 | 胃苏颗粒 | Weisu Granule | 0 |
| 13 | 脂肪乳注射液 | Fat Emulsion Injection | 5 |
| 13 | 艾司唑仑片 | Estazolam Tablets | 0 |
| 13 | 苁蓉通便口服液 | CongRongTong oral liquid | 0 |
| 13 | 苯妥英钠片 | Phenytoin Sodium Tablets | 3 |
| 13 | 辛伐他汀片 | Simvastatin Tablets | 1 |
| 13 | 通窍鼻炎颗粒 | Tong Qiao rhinitis granules | 0 |
| 13 | 酚麻美敏片 | Compound Dextromethorphan Hydrobromide Tablets | 1 |
| 13 | 酪酸梭菌二联活菌散 | Combined Clostridium Butyricum and Bifidobacterium Powders Live | 2 |
| 13 | 酮洛芬凝胶 | Ketoprofen Gel | 1 |
| 13 | 重组人干扰素α1b注射液 | Recombinant Human Interferon α1b Injection | 2 |
| 13 | 阿加曲班注射液 | Argatroban Injection | 0 |
| 13 | 雷贝拉唑钠肠溶胶囊 | Rabeprazole Sodium Enteric-coated Capsules | 0 |
| 13 | 马来酸氯苯那敏片 | Chlorphenamine Maleate Tablets | 0 |
| 14 | 20％甘露醇注射液 | 20% Mannitol Injection | 0 |
| 14 | 地西泮注射液 | Diazepam Injection | 3 |
| 14 | 尼莫地平注射液 | Nimodipine Injection | 0 |
| 14 | 曲克芦丁脑蛋白水解物注射液 | Troxerutin and Cerebroprotein Hydrolysate Injection | 0 |
| 14 | 注射用丙戊酸钠 | Sodium Valproate for Injection | 3 |
| 14 | 注射用单唾液酸四己糖神经节苷脂钠 | Monosialotetrahexosylganglioside Sodium for Injection | 2 |
| 14 | 注射用鼠神经生长因子 | Mouse Nerve Growth Factor for Injection | 0 |
| 14 | 甘油果糖氯化钠注射液 | Glycerol Fructose and Sodium Chloride Injection | 0 |
| 14 | 盐酸氯丙嗪注射液 | Chlorpromazine Hydrochloride Injection | 0 |
| 14 | 盐酸肾上腺素注射液 | Adrenaline Hydrochloride Injection | 0 |
| 14 | 维生素B6注射液 | Vitamin B6 Injection | 0 |
| 14 | 胞磷胆碱钠胶囊 | Citicoline Sodium Capsules | 2 |
| 14 | 脑苷肌肽注射液 | Cattle Encephalon Glycoside and Ignotin Injection | 3 |
| 14 | 脑蛋白水解物口服液 | Cerebroprotein Hydrolysate Oral Solution | 0 |
| 14 | 蔗糖铁注射液 | Iron sucrose Injection | 2 |
| 15 | (婴)维生素AD滴剂 | (baby) Vitamin A and D Drops | 2 |
| 15 | 10％葡萄糖注射液 | 10% Glucose Injection | 2 |
| 15 | 10％葡萄糖酸钙注射液 | 10% Calcium Gluconate Injection | 0 |
| 15 | 25％硫酸镁注射液 | 25% Magnesium Sulfate Injection | 2 |
| 15 | ω-3鱼油脂肪乳注射液 | Fish Oil Fat Emulison Injection | 1 |
| 15 | 中长链脂肪乳注射液 | Medium and Long Chain Fat Emulsion Injection | 0 |
| 15 | 复方托吡卡胺滴眼液 | Compound Tropicamide Eye Drops | 2 |
| 15 | 多种微量元素注射液Ⅱ | Multi-Trace Elements Injection (II) | 0 |
| 15 | 小儿复方氨基酸注射液(19AA-1) | Pediatric Compound Amino Acid Injection(19AA-Ⅰ) | 0 |
| 15 | 枸橼酸咖啡因注射液 | Citrate Caffeine Injection | 5 |
| 15 | 氟康唑氯化钠注射液 | Fluconazole and Sodium Chloride Injection | 0 |
| 15 | 氟康唑胶囊 | Fluconazole Capsules | 0 |
| 15 | 氧氟沙星眼膏 | Ofloxacin Eye Ointment | 2 |
| 15 | 注射用拉氧头孢钠 | Latamoxef Sodium for Injection | 3 |
| 15 | 注射用水溶性维生素 | Water-soluble Vitamin for Injection | 4 |
| 15 | 猪肺磷脂注射液 | Poractant Alfa Injection | 5 |
| 15 | 甘油磷酸钠注射液 | Sodium Glycerophosphate Injection | 0 |
| 15 | 盐酸丙美卡因滴眼液 | Proparacaine Hydrochloride Eye Drops | 0 |
| 15 | 脂溶性维生素注射液Ⅱ | Fat-soluble Vitamin For Injection (II) | 2 |
| 15 | 葡萄糖酸钙口服溶液 | Calcium Gluconate Oral Solution | 0 |
| 15 | 蛋白琥珀酸铁口服溶液 | Iron Proteinsuccinylate Oral Solution | 2 |
| 16 | 5％葡萄糖注射液 | 5% Glucose Injection | 2 |
| 16 | 乌苯美司胶囊 | Ubenimex Capsules | 0 |
| 16 | 依托泊苷注射液 | Etoposide Injection | 2 |
| 16 | 卡铂注射液 | Carboplatin Injection | 0 |
| 16 | 注射用异环磷酰胺 | Ifosfamide for Injection | 2 |
| 16 | 注射用氨磷汀 | Amifostine for Injection | 0 |
| 16 | 注射用环磷酰胺 | Cyclophosphamide for Injection | 0 |
| 16 | 注射用盐酸吡柔比星 | Pirarubicin Hydrochloride for Injection | 2 |
| 16 | 注射用硫酸长春地辛 | Vindesine Sulfate for Injection | 0 |
| 16 | 注射用顺铂 | Cisplatin for Injection | 0 |
| 16 | 盐酸昂丹司琼注射液 | Ondansetron Hydrochloride Injection | 3 |
| 16 | 美司钠注射液 | Mesna Injection | 2 |
| 16 | 肝素钠注射液 | Heparin Sodium Injection | 2 |
| 16 | 胎盘多肽注射液 | Placenta Polypeptide Injection | 0 |
| 17 | 丙戊酸钠口服溶液 | Sodium Valproate Oral Solution | 2 |
| 17 | 丙戊酸钠片 | Sodium Valproate Tablets | 2 |
| 17 | 丙戊酸钠缓释片 | Compound Sodium Valproate and Valproic Acid Sustained Release Tablets | 2 |
| 17 | 卡马西平片 | Carbamazepine Tablets | 5 |
| 17 | 奥卡西平口服混悬液 | Oxcarbazepine Oral Suspension | 2 |
| 17 | 奥卡西平片 | Oxcarbazepine Tablets | 2 |
| 17 | 奥拉西坦胶囊 | Oxiracetam Capsules | 0 |
| 17 | 左乙拉西坦片 | Levetiracetam Tablets | 2 |
| 17 | 托吡酯片 | Topiramate Tablets | 2 |
| 17 | 拉莫三嗪片 | Lamotrigine Tablets | 1 |
| 17 | 氯硝西泮片 | Clonazepam Tablets | 2 |
| 17 | 硝西泮片 | Clonazepan Tablets | 0 |
| 17 | 苯巴比妥片 | Phenobarbital Tablets | 2 |
| 18 | 丁酸氢化可的松乳膏 | Hydrocortisone Butyrate Cream | 2 |
| 18 | 三磷酸腺苷二钠注射液 | Adenosine Disodium Triphosphate Injection | 0 |
| 18 | 二巯丙磺钠注射液 | Sodium Dimercaptopropane Sulfonate Injection | 0 |
| 18 | 亚甲蓝注射液 | Methylthioninium Chloride Injection | 2 |
| 18 | 人凝血因子Ⅷ | Human Coagulation Factor VIII | 0 |
| 18 | 依地酸钙钠注射液 | Cacium Disodium Edetate Injection | 2 |
| 18 | 凝血酶冻干粉 | Lyophilizing Thrombin Powder | 0 |
| 18 | 利妥昔单抗注射液 | Rituximab Injection | 0 |
| 18 | 利福平胶囊 | Rifampicin Capsules | 2 |
| 18 | 华法林钠片 | Warfarin Sodium Tablets | 2 |
| 18 | 卡巴胆碱注射液 | Carbachol Injection | 0 |
| 18 | 卡维地洛片 | Carvedilol Tablets | 0 |
| 18 | 去乙酰毛花苷注射液 | Deslanoside Injection | 5 |
| 18 | 双氯芬酸钠肠溶片 | Diclofenac Sodium Enteric-coated Tablets | 3 |
| 18 | 吡嗪酰胺片 | Pyrazinamide Tablets | 0 |
| 18 | 吸入用硫酸沙丁胺醇溶液 | Salbutamol Sulfate Solution for Inhalation | 3 |
| 18 | 呋喃唑酮片 | Furazolidone Tablets | 3 |
| 18 | 呋喃妥因肠溶片 | Nitrofurantoin Enteric-coated Tablets | 3 |
| 18 | 咪达唑仑注射液 | Midazolam Injection | 0 |
| 18 | 地特胰岛素注射液 | Insulin Detemir Injection | 2 |
| 18 | 地高辛片 | Digoxin Tablets | 5 |
| 18 | 地高辛酏剂 | Digoxin Elixir | 0 |
| 18 | 垂体后叶注射液 | Posterior Pituitary Injection | 0 |
| 18 | 塞来昔布胶囊 | Celecoxib Capsules | 0 |
| 18 | 复方倍他米松注射液 | Compound Betamethasone Injection | 0 |
| 18 | 复方对乙酰氨基酚片Ⅱ | Compound Paracetamol Tablets（Ⅱ） | 2 |
| 18 | 复方氨基酸注射液 | Compound Amino Acid Injection | 0 |
| 18 | 复方泛影葡胺注射液 | Compound　Meglumine　Diatrizoate　Injection | 0 |
| 18 | 复方甘草酸苷注射液 | Compound Glycyrrhizin Injection | 0 |
| 18 | 复方磺胺甲恶唑片 | Compound Sulfamethoxazole Tablets | 3 |
| 18 | 复方胃蛋白酶散 | Compound Pepsin Powder | 2 |
| 18 | 复方芦丁片 | Compound Rutin Tablets | 0 |
| 18 | 复方金钱草颗粒 | Fufang jinqiancao granules | 0 |
| 18 | 复方铁锌口服溶液 | Oral solution of compound iron and zinc | 2 |
| 18 | 头孢呋辛酯片 | Cefuroxime Axetil Tablets | 2 |
| 18 | 妥布霉素地塞米松滴眼液 | Tobramycin and Dexamethasone Eye Drops | 2 |
| 18 | 妥布霉素地塞米松眼膏 | Tobramycin and Dexamethasone Ophthalmic Ointment | 2 |
| 18 | 妥洛特罗贴剂 | Tulobuterol Patch | 3 |
| 18 | 富马酸依美斯汀滴眼液 | Emedastine Difumarate Eye Drops | 2 |
| 18 | 小儿布洛芬栓 | Paediatric Ibuprofen Suppositories | 2 |
| 18 | 尼莫地平片 | Nimodipine Tablets | 0 |
| 18 | 左卡尼汀口服溶液 | Levocarnitine Oral Solution | 2 |
| 18 | 左旋多巴片 | Ethambutol Hydrochloride Tablets | 0 |
| 18 | 左氧氟沙星氯化钠注射液 | Levofloxacin and Sodium Chloride Injection | 0 |
| 18 | 左氧氟沙星滴眼液 | Levofloxacin Eye Drops | 3 |
| 18 | 左甲状腺素钠片 | Levothyroxine Sodium Tablets | 3 |
| 18 | 巴氯芬片 | Baclofen Tablets | 2 |
| 18 | 普拉洛芬滴眼液 | Pranoprofen Eye Drops | 2 |
| 18 | 柳氮磺吡啶肠溶片 | Sulfasalazine Enteric-coated Tablets | 2 |
| 18 | 标准桃金娘油肠溶胶囊 | Myrtol Standardized Enteric Coated Soft Caosules | 2 |
| 18 | 氟哌啶醇片 | Haloperidol Tablets | 2 |
| 18 | 氟米龙滴眼液 | Fluorometholone Eye Drops | 2 |
| 18 | 氧氟沙星凝胶 | Ofloxacin Gel | 0 |
| 18 | 氨茶碱注射液 | Aminophylline Injection | 3 |
| 18 | 沙利度胺片 | Thalidomide Tablets | 0 |
| 18 | 注射用A群链球菌 | Streptococcus A Group For Injection | 0 |
| 18 | 注射用三氧化二砷 | Arsenic Trioxide for Injection | 2 |
| 18 | 注射用促皮质素 | Adernocorticotropime for Injection | 0 |
| 18 | 注射用克林霉素磷酸酯 | Clindamycin Phosphate for Injection | 3 |
| 18 | 注射用尿激酶 | Urokinase for Injection | 0 |
| 18 | 注射用放线菌素D | Dactinomycin for Injection | 3 |
| 18 | 注射用更昔洛韦 | Ganciclovir for Injection | 2 |
| 18 | 注射用氢化可的松琥珀酸钠 | Hydrocortisone Sodium Succinate for Injection | 0 |
| 18 | 注射用氨苄西林钠 | Ampicillin Sodium for Injection | 0 |
| 18 | 注射用生长抑素 | Somatostatin For Injection | 0 |
| 18 | 注射用盐酸万古霉素 | Vancomycin Hydrochloride for Injection | 5 |
| 18 | 注射用盐酸博来霉素 | Bleomycin Hydrochlorid for Injection | 0 |
| 18 | 注射用盐酸博莱霉素 | Bleomycin Hydrochloride for Injection | 0 |
| 18 | 注射用盐酸托泊替康 | Tetracycline Hydrochloride Capsules | 0 |
| 18 | 注射用绒促性素 | Chorionic Gonadotrophin for Injection | 2 |
| 18 | 注射用苄星青霉素 | Benzathine Benzylpenicillin for Injection | 0 |
| 18 | 注射用苯唑西林钠 | Oxacillin Sodium for Injection | 4 |
| 18 | 注射用英夫利西单抗 | Infliximab for Injection | 2 |
| 18 | 注射用达卡巴嗪 | Dacarbazine for Injection | 0 |
| 18 | 注射用重组人Ⅱ型肿瘤坏死因子受体-抗体融合蛋白 | Recombinant Human Tumor Necrosis Factor-αReceptorⅡ:IgG Fc Fusion Protein for Injection | 0 |
| 18 | 注射用重组人凝血因子Ⅷ | Recombinant Coagulation FactorVIII for Injection | 4 |
| 18 | 注射用重组人生长激素 | Recombinant Human Growth Hormone for Injection | 2 |
| 18 | 注射用阿洛西林钠 | Azlocillin sodium for lnjection | 0 |
| 18 | 注射用青霉素钠 | Benzylpenicillin Sodium for Injection | 0 |
| 18 | 浓氯化钠注射液(10％) | 10% Sodium Chloride Injection | 2 |
| 18 | 消旋卡多曲颗粒 | Racecadotril Granules | 3 |
| 18 | 溴吡斯的明片 | Pyridostigmine Bromide Tablets | 0 |
| 18 | 溴芬酸钠水合物滴眼液 | Bromfenac Sodium Hydrate Ophthalmic Solution | 0 |
| 18 | 溴芬酸钠滴眼液 | Bromfenac Sodium Eye Drops | 0 |
| 18 | 甘精胰岛素注射液 | Insulin Glargine Injection | 0 |
| 18 | 甲巯咪唑片 | Methimazole Tablets | 2 |
| 18 | 甲硝唑片 | Metronidazole Tablets | 2 |
| 18 | 甲硫酸新斯的明注射液 | NEOSTIGMINE METHYLSULFATE INJECTION | 0 |
| 18 | 甲苯咪唑片 | Mebendazole Tablets | 3 |
| 18 | 甲钴胺分散片 | Methylcobalamin Dispersible Tablets | 2 |
| 18 | 甲钴胺注射液 | Mecobalamin Injection | 0 |
| 18 | 盐酸二甲双胍片 | Metformin Hydrochloride Tablets | 0 |
| 18 | 盐酸异丙嗪注射液 | Promethazine Hydrochloride Injection | 3 |
| 18 | 盐酸普罗帕酮注射液 | Propafenone Hydrochloride Injection | 0 |
| 18 | 盐酸普罗帕酮片 | Propafenone Hydrochloride Tablets | 0 |
| 18 | 盐酸普萘洛尔片 | Propranolol | 2 |
| 18 | 盐酸氟桂利嗪胶囊 | Flunarizine Hydrochloride Capsules | 0 |
| 18 | 盐酸氯胺酮注射液 | Ketamine Hydrochloride Injection | 2 |
| 18 | 盐酸硫必利片 | Tiapride Hydrochloride Tablets | 2 |
| 18 | 盐酸精氨酸注射液 | Arginine Hydrochloride Injection | 0 |
| 18 | 盐酸胺碘酮注射液 | Amiodarone Hydrochloride injection | 0 |
| 18 | 盐酸胺碘酮片 | Amiodarone Hydrochloride Tablets | 0 |
| 18 | 盐酸舍曲林片 | Sertraline Hydrochloride Tablets | 2 |
| 18 | 盐酸苯海索片 | Benzhexol Hydrochloride Tablets | 0 |
| 18 | 盐酸赛庚啶片 | Cyproheptadine Hydrochloride Tablets | 0 |
| 18 | 破伤风人免疫球蛋白 | Human Tetanus Immunoglobulin | 2 |
| 18 | 破伤风抗毒素注射液 | Tetanus Antitoxin Injection | 4 |
| 18 | 硝酸甘油注射液 | Nitroglycerin Injection | 0 |
| 18 | 硫唑嘌呤片 | Azathioprine Tablets | 0 |
| 18 | 硫糖铝口服混悬液 | Sucralfate Oral Suspension | 2 |
| 18 | 硫酸氢氯吡格雷片 | Clopidogrel Hydrogen Sulphate Tablets | 0 |
| 18 | 硫酸羟氯喹片 | Hydroxychloroquine Sulfate Tablets | 2 |
| 18 | 硫酸阿托品注射液 | Atropine Sulfate Injection | 3 |
| 18 | 硫酸阿托品眼用凝胶 | Atropine Sulfate Eye Gel | 2 |
| 18 | 碘佛醇注射液 | Ioversol Injection | 3 |
| 18 | 碘化油注射液 | Iodinated Oil Injection | 0 |
| 18 | 碘普罗胺注射液 | Iopromide Injection | 4 |
| 18 | 碘海醇注射液 | lohexol lnjectlon | 3 |
| 18 | 磷酸奥司他韦颗粒 | Oseltamivir Phosphate Granules | 3 |
| 18 | 积雪苷霜软膏 | Centella Triterpenes Cream | 2 |
| 18 | 精蛋白生物合成人胰岛素注射 | Isophane Protamine Biosynthetic Human Insulin Injection | 2 |
| 18 | 糠酸莫米松乳膏 | Momeiasone Furoate Cream | 2 |
| 18 | 维A酸片 | Tretinoin Tablets | 0 |
| 18 | 维生素B12注射液 | Vitamin B12 Injection | 5 |
| 18 | 维生素B1片 | Vitamin B1 Tablets | 2 |
| 18 | 维生素B6片 | Vitamin B6 Tablets | 2 |
| 18 | 维生素C注射液 | Vitamin C Injection | 0 |
| 18 | 维生素C片 | Vitamin C Tablets | 2 |
| 18 | 美沙拉秦缓释颗粒 | Mesalazine Sustained Release Granules | 0 |
| 18 | 羚羊角胶囊 | Antelope horn capsule | 0 |
| 18 | 羚羊角颗粒 | Antelope horn granules | 2 |
| 18 | 羟基脲片 | Hydroxyurea Tablets | 0 |
| 18 | 联苯苄唑乳膏 | Bifonazole Cream | 0 |
| 18 | 肠内营养混悬液（TPF-D） | Enteral Nutritional Suspension(TPF-D) | 0 |
| 18 | 肾炎四味胶囊 | ShenYanShiWei Capsule | 0 |
| 18 | 茵栀黄口服液 | Yinzhihuang Oral Liquid | 0 |
| 18 | 茵栀黄颗粒 | YinZhiHuang Granules | 0 |
| 18 | 药用枸橼酸钠 | Sodium Citrate | 0 |
| 18 | 药用枸橼酸钾 | Bismuth Potassium | 2 |
| 18 | 莫匹罗星软膏 | Mupirocin Cream | 0 |
| 18 | 葡萄糖酸锌颗粒 | Zinc Gluconate Granulate | 3 |
| 18 | 葫芦素片 | Calebassine Tablets | 2 |
| 18 | 谷维素片 | Oryzanol Tablets | 0 |
| 18 | 赖氨酸硫酸锌口服溶液 | Lysine Zinc Sulfate Oral Solution | 0 |
| 18 | 赖脯胰岛素注射液 | Recombinant Human Insulin Lispro Injection | 2 |
| 18 | 轻质液状石蜡 | Light Liquid Paraffin | 0 |
| 18 | 酒石酸美托洛尔片 | Metoprolol Tartrate Tablets | 0 |
| 18 | 酪酸梭菌活菌散剂 | Clostridium Butyricum Powders | 0 |
| 18 | 醋酸去氨加压素片 | Desmopressin Acetate Tablets | 2 |
| 18 | 醋酸曲普瑞林注射液 | Triptorelin Acetate Injection | 0 |
| 18 | 醋酸氢化可的松片 | Hydrocortisone Acetate Tablets | 2 |
| 18 | 重组人胰岛素注射液 | Recombinant Human Insulin Injection | 2 |
| 18 | 重酒石酸去甲肾上腺素注射液 | Noradrenaline Bitartrate Injection | 2 |
| 18 | 门冬氨酸钾镁注射液 | Potassium Magnesium Aspartate Injection | 2 |
| 18 | 门冬胰岛素注射液 | Insulin Aspart Injection | 2 |
| 18 | 阿立哌唑片 | Aripiprazole Tablets | 0 |
| 18 | 青霉胺片 | PenicillamineTablets | 0 |
| 18 | 静脉注射用人免疫球蛋白 | Human Immunoglobulin for Intravenous Injection | 0 |
| 18 | 颠茄合剂 | DianQieHeJi | 0 |
